# Improved Egret Swarm Optimization Algorithm Based on Variable-Factor Weighted Learning and Adjacent Generation Dimension Crossover Strategy

**DOI:** 10.3390/biomimetics11060365

**Published:** 2026-05-23

**Authors:** Sunde Wang, Yejun Zheng, Pu Wang, Zihao Cheng

**Affiliations:** 1 School of Electronics and Electrical Engineering, Wenzhou University of Technology, Wenzhou 325035, China; 2 Engineering Technology Department, Shanghai Caoyang Vocational School, Shanghai 200333, China; 3 School of Computer Engineering, Hubei University of Arts and Science, Xiangyang 441053, China; 4 College of Control Science and Engineering, Zhejiang University, Hangzhou 310027, China

**Keywords:** egret swarm optimization algorithm, variable-factor weighted learning, adjacent generation dimension crossover, preferred mutation reverse learning, engineering optimization design

## Abstract

To address the defects of the traditional egret swarm optimization algorithm (ESOA) in high-dimensional complex optimization problems, such as low optimization accuracy, weak ability to escape from local extrema, rapid decay of population diversity, and insufficient efficiency in the late convergence stage, an improved egret swarm optimization algorithm (IESOA) combining variable-factor weighted learning and adjacent generation dimension crossover strategy is proposed. Firstly, a dynamic change rule of core model parameters (exploration factor ω and exploitation factor μ) is constructed to adaptively adjust with the iteration process, so as to balance global exploration and local exploitation capabilities. Secondly, a multi-individual variable-factor weighted learning mechanism is designed to enable offspring individuals to inherit the position information of following individuals, sub-population optimal individuals, and global optimal individuals simultaneously, avoiding excessively fast assimilation of the population. Furthermore, an adjacent generation dimension crossover strategy is established to update the optimal individual based on the priority principle of absolute dimension difference, fully retaining the historical optimal dimension information. Finally, a preferred mutation reverse learning strategy is integrated to further enhance the local extremum escape ability and convergence accuracy of the algorithm. The IESOA is compared with eight algorithms, including PSO, DE, SBOA, BKA, HHO, DOA, and the original ESOA on CEC2014 and CEC2019 benchmark test suites. The results show that IESOA presents significant advantages in optimization accuracy, convergence speed, and stability. The algorithm is applied to three typical engineering optimization problems: reinforced concrete beam design, welded beam design, and pressure vessel design, which effectively reduces the structural design cost and verifies its application value in practical engineering.

## 1. Introduction

Intelligent optimization algorithms are a class of stochastic optimization methods inspired by natural phenomena, biological group behaviors, or physical laws. Benefiting from the advantages of insensitivity to initial values, concise principles, strong robustness, and independence from mathematical characteristics of problems, global intelligent optimization algorithms have been widely applied in practical engineering scenarios, including intelligent manufacturing production scheduling, transportation path planning, aerospace structural optimization, energy system parameter tuning, civil engineering design, robot trajectory optimization, new energy allocation scheduling, and parameter identification of complex constrained industrial systems. They show irreplaceable application value in complex optimization problems with high dimension, nonlinearity, multi-constraint, and non-convex characteristics, where traditional gradient-based algorithms are difficult to apply. Meanwhile, they have been extensively promoted and practiced in academic and engineering fields such as supply chain network optimization [1], system-level fault diagnosis [2], production scheduling [3], and engineering structural design [4]. According to different heuristic mechanisms, intelligent optimization algorithms can be divided into three categories. The first category is evolutionary-based algorithms, such as Genetic Algorithm (GA) [5] and Evolutionary Programming [6], which realize population iterative updating by simulating the selection, crossover, and mutation of biological genes. The second category is swarm intelligence algorithms based on biological cooperative behavior, such as Particle Swarm Optimization (PSO) [7], Ant Colony Optimization (ACO) [8], and Crow Search Algorithm (CSA) [9], which complete optimization by simulating group foraging and migration behaviors. The third category is physics-inspired algorithms, such as Simulated Annealing (SA) [10], Multi-Verse Optimizer (MVO) [11], and Search Group Algorithm (SGA) [12], which obtain optimization capability by mathematically modeling physical processes.

With the continuous development of engineering technology, practical optimization problems gradually present complex characteristics such as high dimension, strong coupling, and multiple constraints, which put forward higher requirements on the convergence speed, optimization accuracy, population diversity, and local optimum escape ability of intelligent optimization algorithms. The egret swarm optimization algorithm (ESOA) is a novel swarm intelligence algorithm proposed by Chen et al. in 2022 [13]. Inspired by the aggressive strategy of great egrets and the sit-and-wait strategy of snow egrets, it divides the population into multiple subgroups and completes optimization through cooperative search of different individuals. With simple parameter setting and fast convergence speed, ESOA has been initially applied in mobile robot path planning [14], manipulator trajectory planning [15], boiler combustion optimization [16], and other fields. However, when solving high-dimensional complex optimization problems, traditional ESOA exposes many limitations: first, the core parameters (exploration factor and exploitation factor) adopt fixed values, which cannot dynamically balance global exploration and local exploitation with iteration progress, resulting in insufficient global search in the early stage or inadequate local exploitation in the later stage. Second, offspring individuals only update their positions by learning from a single follower, which easily causes rapid population assimilation, reduces population diversity, and increases the risk of falling into local optima. Third, the update of the optimal individual only depends on the current optimal solution, without fully utilizing the dimensional information of historical optimal individuals, leading to a weak ability to escape local optima. Fourth, the convergence speed slows down in the later iterations, and the optimization accuracy has difficulty meeting the requirements of complex engineering problems [17,18]. These shortcomings seriously restrict the application effects of the ESOA in high-dimensional complex engineering optimization scenarios. First, the core model parameters (exploration factor and exploitation factor) adopt fixed values, which cannot dynamically adjust the balance between global exploration and local exploitation according to the iteration process, leading to insufficient global search in the early stage or inadequate local exploitation in the later stage. Second, offspring individuals update their positions only by learning from a single individual (following individual), which easily leads to excessively fast assimilation of the population, reduces population diversity, and increases the risk of falling into local extrema [19]. Third, the update of optimal individuals only relies on the current iterative optimal solution and fails to make full use of the dimension information of historical optimal individuals, resulting in weak local extremum escape ability. Fourth, the convergence speed slows down in the later stage of the algorithm, and the optimization accuracy has difficulty meeting the requirements of complex engineering problems. These defects seriously limit the application effects of the ESOA in high-dimensional complex engineering optimization scenarios [20].

Based on the above improvement ideas, this paper proposes an improved egret swarm optimization algorithm (IESOA) for the inherent limitations of traditional ESOA. The optimization performance of the algorithm is comprehensively improved by designing dynamic change rules of model parameters, constructing a multi-individual variable-factor weighted learning mechanism, and introducing an adjacent generation dimension crossover strategy and a preferred mutation reverse learning strategy. The superiority of the IESOA in optimization problems of different dimensions and types is verified through the CEC2014 and CEC2019 benchmark test functions. The algorithm is applied to three typical engineering optimization problems: reinforced concrete beam design, welded beam design, and pressure vessel design, providing a new and effective method for the optimal design of complex engineering structures.

Aiming at the inherent drawbacks of traditional ESOA, such as fixed parameter setting, easy loss of population diversity, weak local optimum escape ability, and insufficient optimization accuracy in high-dimensional problems, this paper proposes an improved egret swarm optimization algorithm (IESOA). The main contributions of this paper are summarized as follows:

(1) A dynamic variation rule of multiple parameters is designed to break the fixed parameter pattern of a traditional ESOA, realizing adaptive balance between global exploration and local exploitation with iterative progress, and effectively overcoming insufficient early search and weak convergence later;
(2) A multi-individual variable-factor weighted learning mechanism is constructed, combined with adjacent generation dimension crossover and optimal-guided mutation opposition-based learning strategy, which enriches population updating modes, delays premature convergence, and strengthens the algorithm’s ability to jump out of local optima and global optimization potential;
(3) Comprehensive performance validation is conducted via the CEC2014 and CEC2019 benchmark functions with multiple dimensions. The proposed IESOA is further applied to typical constrained engineering optimization problems, including reinforced concrete beam design, welded beam design, and pressure vessel design, providing a new efficient alternative method for similar structural engineering optimization.

## 2. Egret Swarm Optimization Algorithm (ESOA)

The ESOA simulates the cooperative predation behavior of egret swarms. The population is divided into several egret sub-populations, and each sub-population contains three egrets (Egret A, Egret B, and Egret C) that perform different search strategies [21]. The iterative update of the population is realized through discriminant conditions. Its core mechanisms include three parts: the sit-and-wait strategy, the attack strategy, and the discriminant condition. Suppose that in a *d*-dimensional optimization problem, the egret population size is *N*, and the position of the *i*-th sub-population at the *t*-th iteration is $x^{i,t}=\left[x_{1}^{i,t},x_{2}^{i,t},\ldots ,x_{d}^{i,t}\right]$, where i=1,2,⋯,N, $t=1,2,\cdots ,t_{max}$, and $t_{max}$ is the maximum number of iterations. Each sub-population memorizes its own historical optimal position $x_{ibest}^{t}$ (the optimal position of the *i*-th sub-population at the *t*-th iteration) and the global optimal position of the population $x_{gbest}^{t}$ (the optimal position of the whole population at the *t*-th iteration). The fitness function is $f\left(\cdot \right)$, which is used to evaluate the quality of individual positions [22].

### 2.1. Sit-and-Wait Strategy (Egret A)

Egret A simulates the “sit-and-wait strategy” of snowy egrets and performs local search through pseudo-gradient estimation to achieve accurate predation with low energy consumption. The position update formula is:

(1) $$x_{a,i}^{t+1}=x_{i}^{t}+step_{s}\cdot exp\left(-\frac{t}{0.1\cdot t_{max}}\right)\cdot hop\cdot g_{i}^{t}$$

where $x_{a,i}^{t+1}$ is the position of Egret A in the *i*-th sub-population at the (*t* + 1)-th iteration. $step_{s}\in (0,1]$ is the step factor. *hop* = *u_b_* − *l_b_* is the difference between the upper and lower bounds of the solution space (*u_b_* and *l_b_* are the upper and lower bounds of the solution space, respectively). $g_{i}^{t}=\left(f\left(x_{i}^{t}\right)-f\left(x_{gbest}^{t}\right)\right)\cdot x_{i}^{t}$ is the pseudo-gradient vector, reflecting the gradient difference between the current position and the global optimal position. $f\left(x_{i}^{t}\right)$ is the fitness value of the current position. $f\left(x_{gbest}^{t}\right)$ is the fitness value of the global optimal position [23].

### 2.2. Attack Strategy (Egret B and Egret C)

Egret B simulates the random search behavior of great egrets and adopts a random walk strategy for global exploration to expand the search range. The position update formula is:

(2) $$x_{b,i}^{t+1}=x_{i}^{t}+step_{b}\cdot tan\left(r_{b,i}\right)\cdot \frac{hop}{1+t}$$

where $x_{b,i}^{t+1}$ is the position of Egret B in the *i*-th sub-population at the (*t* + 1)-th iteration. $step_{b}\in \left(0,1\right]$ is the step factor. $r_{b,i}\in \left(-\pi /2,\;\pi /2\right)$ is the random direction angle, used to generate random moving directions. t is the current number of iterations, and the step size decreases through $\frac{hop}{1+t}$ to balance early exploration and later exploitation [24].

Egret C simulates the encircling predation behavior of great egrets and adopts an encircling mechanism for local exploitation, approaching the sub-population optimal and global optimal positions. The position update formula is:

(3) $$x_{c,i}^{t+1}=\left(1-r_{h}-r_{g}\right)\cdot x_{i}^{t}+r_{h}\cdot D_{h}^{t}+r_{g}\cdot D_{g}^{t}$$

where $x_{c,i}^{t+1}$ is the position of Egret C in the *i*-th sub-population at the (*t* + 1)-th iteration. $r_{h},r_{g}\in \left[0,0.5\right)$ are random coefficients, used to balance the influence of individual position and optimal position. $D_{h}^{t}=x_{ibest}^{t}-x_{i}^{t}$ is the difference between the current position and the sub-population optimal position. $D_{g}^{t}=x_{gbest}^{t}-x_{i}^{t}$ is the difference between the current position and the global optimal position [25].

### 2.3. Discriminant Condition

After each iteration, the egret sub-population selects the optimal solution to update the sub-population position by comparing the fitness values obtained by the three strategies. The specific rules are [26]:

(4) $$x_{s,i}^{t+1}=\left[x_{a,i}^{t+1},{\;x}_{b,i}^{t+1},\;x_{c,i}^{t+1}\right]$$

(5) $$y_{s,i}^{t+1}=\left[f\left(x_{a,i}^{t+1}\right),\;f\left(x_{b,i}^{t+1}\right),\;f\left(x_{c,i}^{t+1}\right)\right]$$

(6) $$c_{i}=argmin\left(y_{s,i}^{t+1}\right)$$

(7) $$x_{i}^{t+1}=\left\{\begin{array}{ll} x_{s,i}^{t+1}|\;c_{i} & \;f(xs,i^{t+1}{|}_{c_{i}})<f(x_{i}^{t})\;\mathrm{or}\;r<0.3\\ x_{i}^{t} & \;\mathrm{others} \end{array}\right.$$

where $x_{s,i}^{t+1}$ is the candidate position set generated by the three strategies. $y_{s,i}^{t+1}$ is the fitness value set corresponding to the candidate positions. $c_{i}$ is the index corresponding to the optimal fitness value. $r\in \left(0,1\right)$ is a random number, and a 30% probability is set to accept inferior positions to increase population diversity and avoid the algorithm falling into local optima [27].

During the iteration, the historical optimal position of the sub-population and the global optimal position of the population are continuously updated:

(8) $$x_{ibest}^{t+1}=\left\{\begin{array}{ll} x_{i}^{t+1} & \;f(x_{i}^{t+1})<f(x_{ibest}^{t})\\ x_{ibest}^{t} & \;\mathrm{others} \end{array}\right.$$

(9) $$x_{gbest}^{t+1}=argmin\left[f\left(x_{1best}^{t+1}\right),f\left(x_{2best}^{t+1}\right),\ldots ,f\left(x_{Nbest}^{t+1}\right)\right]$$

Although the original ESOA exhibits competitive performance on low-dimensional simple optimization problems, its inherent limitations become prominent when solving high-dimensional complex tasks, which are summarized in four aspects [28]:

(1) Fixed parameter setting, lack of dynamic adaptability: The core parameters of the ESOA ($step_{s}$, $step_{b}$, $r_{h}$, $r_{g}$) are all fixed values and cannot be dynamically adjusted according to the iteration process. Strong global exploration ability is required in the early iteration to traverse the solution space, while fixed parameters make it difficult to ensure sufficient global search. Strong local exploitation ability is required in the later iterations to approach the optimal solution, while fixed parameters may lead to insufficient exploitation or excessive exploration, affecting the convergence accuracy.
(2) Single-source learning mechanism leading to insufficient population diversity. The position updating rules of Egret B and Egret C merely depend on a single information source, including the current individual, sub-population optimum, or global optimum. Such a single-individual learning pattern accelerates population homogenization, reduces population diversity, and increases the risk of stagnation in local optima.
(3) Defective elite individual updating strategy. The optimal individuals are updated merely based on the current iteration optimum, ignoring valuable dimensional information from historical elite solutions. In practice, different historical optima may contain high-quality dimensional variables that are close to the global optimum. Neglecting such historical information weakens the algorithm’s ability to escape local extremum [29].
(4) Low convergence efficiency and limited optimization precision in the late stage. As iterations proceed, population individuals gradually converge toward local optima with continuously decreasing search steps. This phenomenon results in slow late-stage convergence, difficulty in approaching the global optimum, and insufficient optimization accuracy for high-dimensional complex engineering problems.

## 3. Improved Egret Swarm Optimization Algorithm with Variable-Factor Weighted Learning and Adjacent Generation Dimension Crossover Strategy (IESOA)

To solve the above limitations of the traditional ESOA, this paper proposes an improved egret swarm optimization algorithm (IESOA). The optimization performance of the algorithm is comprehensively improved through four strategies: dynamic change in model parameters, multi-individual variable-factor weighted learning, adjacent generation dimension crossover, and preferred mutation reverse learning.

### 3.1. Dynamic Change Rule of Model Parameters

The core parameters of the ESOA (exploration factor *ω* and exploitation factor *μ*) directly affect the global exploration and local exploitation capabilities of the algorithm [30]. The exploration factor *ω* controls the random walk step size of Egret B, determining the global search range. The exploitation factor μ controls the weight of the encircling mechanism of Egret C, determining the local search accuracy. To achieve the dynamic balance between exploration and exploitation capabilities during the iteration, a dynamic change rule is designed where the parameters decrease with the iteration process: the parameter values are large in the early iteration to ensure strong global exploration ability, and gradually decrease in the later iteration to enhance local exploitation ability until approaching the preset minimum value [31].

**(1) Dynamic change in exploration factor ω**

The exploration factor *ω* is used to adjust the random walk step size of Egret B. Its dynamic change function is designed in three forms: linear decreasing function (*LDF*), concave decreasing function (*CaDF*), and convex decreasing function (*CvDF*):

(10) $$\omega^{LDF}(t)=\omega_{max}-t\times \frac{\omega_{max}-\omega_{min}}{t_{max}}$$

(11) $$\omega^{CaDF}(t)=(\omega_{max}-\omega_{min})\times {\left(1-{\left(\frac{t}{t_{max}}\right)}^{\alpha }\right)}^{1/\alpha }+\omega_{min}$$

(12) $$\omega^{CvDF}(t)=(\omega_{max}-\omega_{min})\times {\left(\frac{t}{t_{max}}\right)}^{\alpha }+\omega_{min}$$

where $\omega_{max}$ and $\omega_{min}$ are the maximum and minimum values of the exploration factor, respectively. In this paper, $\omega_{max}=0.9$ and $\omega_{min}=0.1$ are set. α>1 is the shape parameter, and α=2 is adopted in this paper. t is the current number of iterations, and $t_{max}$ is the maximum number of iterations.

**(2) Dynamic change in exploitation factor *μ***

The exploitation factor *μ* is used to adjust the weight of the encircling mechanism of Egret C. Its dynamic change function is similar to that of the exploration factor, also designed in three forms:

(13) $$\mu^{LDF}(t)=\mu_{max}-t\times \frac{\mu_{max}-\mu_{min}}{t_{max}}$$

(14) $$\mu^{CaDF}(t)=(\mu_{max}-\mu_{min})\times {\left(1-{\left(\frac{t}{t_{max}}\right)}^{\alpha }\right)}^{1/\alpha }+\mu_{min}$$

(15) $$\mu^{CvDF}(t)=(\mu_{max}-\mu_{min})\times {\left(\frac{t}{t_{max}}\right)}^{\alpha }+\mu_{min}$$

where $\mu_{max}$ and $\mu_{min}$ are the maximum and minimum values of the exploitation factor, respectively. In this paper, $\mu_{max}=0.8$ and $\mu_{min}=0.2$ are set, and α=2 is the shape parameter.

Through the dynamic change rule, the exploration factor *ω* and exploitation factor μ are adaptively adjusted with the iteration process, realizing the dynamic balance between global exploration and local exploitation, and avoiding the problems of insufficient exploration or excessive exploitation caused by fixed parameters.

### 3.2. Multi-Individual-Based Variable-Factor Weighted Learning Strategy

To solve the problem of insufficient population diversity caused by single-individual learning in the traditional ESOA, a multi-individual variable-factor weighted learning mechanism is introduced. Offspring individuals inherit the position information of the following individuals, sub-population optimal individuals, and global optimal individuals simultaneously. The weighted fusion realizes the effective utilization of multi-source information, ensures population diversity, and reduces the risk of falling into local extrema [32,33].

Suppose that at the *t*-th iteration of the *i*-th sub-population, the following individual is the *j*-th sub-population (j≠i) with position $x_{j}^{t}$, the sub-population optimal position is $x_{ibest}^{t}$, and the global optimal position of the population is $x_{gbest}^{t}$. Under the variable-factor weighted learning mechanism, the position update formulas of Egret B and Egret C are reconstructed as follows [34]:

(1) Position update of Egret B (global exploration):

(16) $$x_{b,i}^{t+1}=x_{i}^{t}+\omega (t)\times \tan(r_{b,i})\times \frac{hop}{1+t}\times \left(\lambda (t)\times x_{j}^{t}+(1-\lambda (t))\times x_{gbest}^{t}-x_{i}^{t}\right)$$

where $\lambda \left(t\right)$ is the weighted learning factor, used to balance the information contribution of the following individuals and global optimal individuals. Its dynamic change rule is consistent with parameter $\omega \left(t\right)$, designed in three forms: *LDF*, *CaDF* and *CvDF*:

(17) $$\lambda^{LDF}(t)=\lambda_{max}-t\times \frac{\lambda_{max}-\lambda_{min}}{t_{max}}$$

(18) $$\lambda^{CaDF}(t)=(\lambda_{max}-\lambda_{min})\times {\left(1-{\left(\frac{t}{t_{max}}\right)}^{\alpha }\right)}^{1/\alpha }+\lambda_{min}$$

(19) $$\lambda^{CvDF}(t)=(\lambda_{max}-\lambda_{min})\times {\left(\frac{t}{t_{max}}\right)}^{\alpha }+\lambda_{min}$$

where $\lambda_{max}=0.9$, $\lambda_{min}=0.1$, and α=2. In the early iteration, $\lambda \left(t\right)$ is large, and offspring individuals inherit more information from following individuals to ensure the global exploration range. In the later iteration, $\lambda \left(t\right)$ is small, and more information from global optimal individuals is inherited to enhance the local exploitation accuracy.

(2) Position update of Egret C (local exploitation):

(20) $$x_{c,i}^{t+1}=(1-\mu (t)\times (r_{h}+r_{g}))\times x_{i}^{t}+\mu (t)\times r_{h}\times x_{ibest}^{t}+\mu (t)\times r_{g}\times x_{gbest}^{t}$$

where $\mu \left(t\right)$ is the dynamic exploitation factor, which adaptively enhances the local exploitation ability by weighted fusion of the position information of current individuals, sub-population optimal individuals, and global optimal individuals. The multi-individual variable-factor weighted learning mechanism effectively expands the search space of offspring individuals through multi-source information fusion, ensures population diversity, and avoids the problem of excessively fast assimilation of the population caused by single-individual learning.

### 3.3. Optimal Individual Update Mechanism Based on Adjacent Generation Dimension Crossover Strategy

To make full use of the dimension information of historical optimal individuals and enhance the local extremum escape ability of the algorithm, an adjacent generation dimension crossover strategy is proposed [35,36]. This strategy compares the dimension differences in optimal individuals in two adjacent generations (the global optimal $x_{gbest}^{t}$ at the *t*-th iteration and the global optimal $x_{gbest}^{t-1}$ at the (*t* − 1)-th iteration), and performs crossover operations based on the principle of priority of maximum absolute dimension difference, retaining the optimal dimension information of all generations to realize accurate update of optimal individuals [37].

(1) Dimension difference calculation and sorting

Calculate the absolute difference in each dimension of optimal individuals in the two adjacent generations:

(21) $$\Delta_{k}^{t}=\left|x_{gbest,k}^{t}-x_{gbest,k}^{t-1}\right|,\;k=1,2,\cdots ,d$$

where $\Delta_{k}^{t}$ is the absolute difference in the k-th dimension. $x_{gbest,k}^{t}$ is the k-th dimension value of the global optimal individual at the t-th iteration. $x_{gbest,k}^{t-1}$ is the *k*-th dimension value of the global optimal individual at the (*t* − 1)-th iteration. d is the problem dimension.

Sort the dimension differences in descending order to obtain the difference sorting index:

(22) $$\left[S\Delta^{t},SInd^{t}]=sort(\Delta^{t}{,}^{\prime }descend^{\prime }\right)$$

where $S\Delta^{t}$ is the sorted dimension difference value, and $SInd^{t}$ is the corresponding dimension index.

(2) Dimension crossover operation

Set the dimension crossover ratio $R_{cross}$ ($R_{cross}=0.3$ in this paper), and the maximum crossover times is $\left\lfloor R_{cross}\cdot d\right\rfloor$. Perform crossover operations on each dimension, in turn, according to the difference sorting index. If the fitness value is better after crossover, retain the crossover result. Otherwise, abandon the crossover. The specific rules are:

(23) $$x_{gbest,k}^{t+1}=\left\{\begin{array}{ll} x_{gbest,k}^{t-1} & \;f(x_{gbest}^{t,\mathrm{cross}})<f(x_{gbest}^{t})\\ x_{gbest,k}^{t} & \;\mathrm{others} \end{array}\right.,\;k=SInd_{1}^{t},SInd_{2}^{t},\cdots ,SInd_{R_{cross}\cdot d}^{t}$$

where ${x^{\prime }}_{gbest}$ is the candidate optimal individual after crossover of the *k*-th dimension. $SInd_{h}^{t}$ is the dimension index corresponding to the *h*-th largest difference ($h=1,\;2,\cdots ,\;\;\lfloor R_{cross}\cdot d\rfloor$).

The adjacent generation dimension crossover strategy effectively enhances the dimension depth mining ability and local extremum escape ability of the algorithm by fusing the high-quality dimension information of historical optimal individuals, avoiding the optimal individual falling into the local optimal neighborhood [38].

### 3.4. Preferred Mutation Reverse Learning Strategy

To further improve the local extremum escape ability and convergence accuracy of the algorithm, a preferred mutation reverse learning strategy is introduced. Based on the idea of reverse learning, this strategy generates reverse mutation individuals of the current optimal individual to expand the local search range [39,40]. Combined with the preferred selection mechanism, individuals with better fitness are retained to realize accurate convergence of the algorithm.

(1) Reverse mutation individual generation

Based on the global optimal individual $x_{gbest}^{t}$ at the *t*-th iteration, generate reverse mutation individuals:

(24) $$x_{rev}^{t}=lb+ub-x_{gbest}^{t}+\sigma \times (rand(1,d)-0.5)$$

where $x_{rev}^{t}$ is the reverse mutation individual. σ is the mutation step factor (σ=0.3 in this paper). $rand\left(1,d\right)$ is a d-dimensional random vector in the [0, 1] interval, used to introduce random mutation characteristics [41].

(2) Preferred selection mechanism

Calculate the fitness value of the reverse mutation individual, compare it with the current global optimal individual, and select the individual with better fitness as the new global optimal individual:

(25) $$x_{gbest}^{t+1}=\left\{\begin{array}{ll} x_{rev}^{t} & \;f(x_{rev}^{t})<f(x_{gbest}^{t})\\ x_{gbest}^{t} & \;\mathrm{others} \end{array}\right.$$

The preferred mutation reverse learning strategy expands the local search range through reverse mutation, ensures the convergence accuracy of the algorithm combined with preferred selection, and effectively avoids the global optimal individual falling into local extrema [42].

### 3.5. Algorithm Complexity Analysis

The algorithm complexity is mainly composed of three parts: initialization, iterative update, and optimal individual update. Time complexity is taken as the main evaluation index, and the specific analysis is as follows:

(1) Initialization stage: Initialize the positions of the egret sub-populations with population size *N*, and the time complexity is $O\left(N\cdot d\right)$. Calculate initial fitness values, and the time complexity is $O\left(N\cdot d\right)$. Initialize the historical optimal of sub-populations and the global optimal position of the population, and the time complexity is $O\left(N\cdot d\right)$. The total time complexity of this stage is $O\left(N\cdot d\right)$.
(2) Iterative update stage: In each iteration, the time complexity of position update operations of Egret A, B, and C is $O\left(N\cdot d\right)$. The time complexity of fitness value calculation is $O\left(N\cdot d\right)$. The time complexity of the sub-population position update and the optimal position update is $O\left(N\cdot d\right)$. The total time complexity of this stage is $O\left(t_{max}\cdot N\cdot d\right)$, where $t_{max}$ is the maximum number of iterations.
(3) Optimal individual optimization stage: The time complexity of dimension difference calculation, sorting, and crossover operations of the adjacent generation dimension crossover strategy is $O\left(dlogd\right)$. The time complexity of reverse individual generation and preferred selection of the preferred mutation reverse learning strategy is $O\left(d\right)$. The total time complexity of this stage is $O\left(t_{max}\cdot dlogd\right)$.

In summary, the total time complexity of the IESOA is $O\left(N\cdot d+t_{max}\cdot N\cdot d+t_{max}\cdot dlogd\right)$. Since $t_{max}$ and *N* are usually constants much larger than *d*, and dlogd is much smaller than N·d, the time complexity of the IESOA can be simplified to $O\left(t_{max}\cdot N\cdot d\right)$, which is consistent with that of the traditional ESOA. This indicates that the improved strategy does not significantly increase the computational overhead of the algorithm.

**Figure 1:**
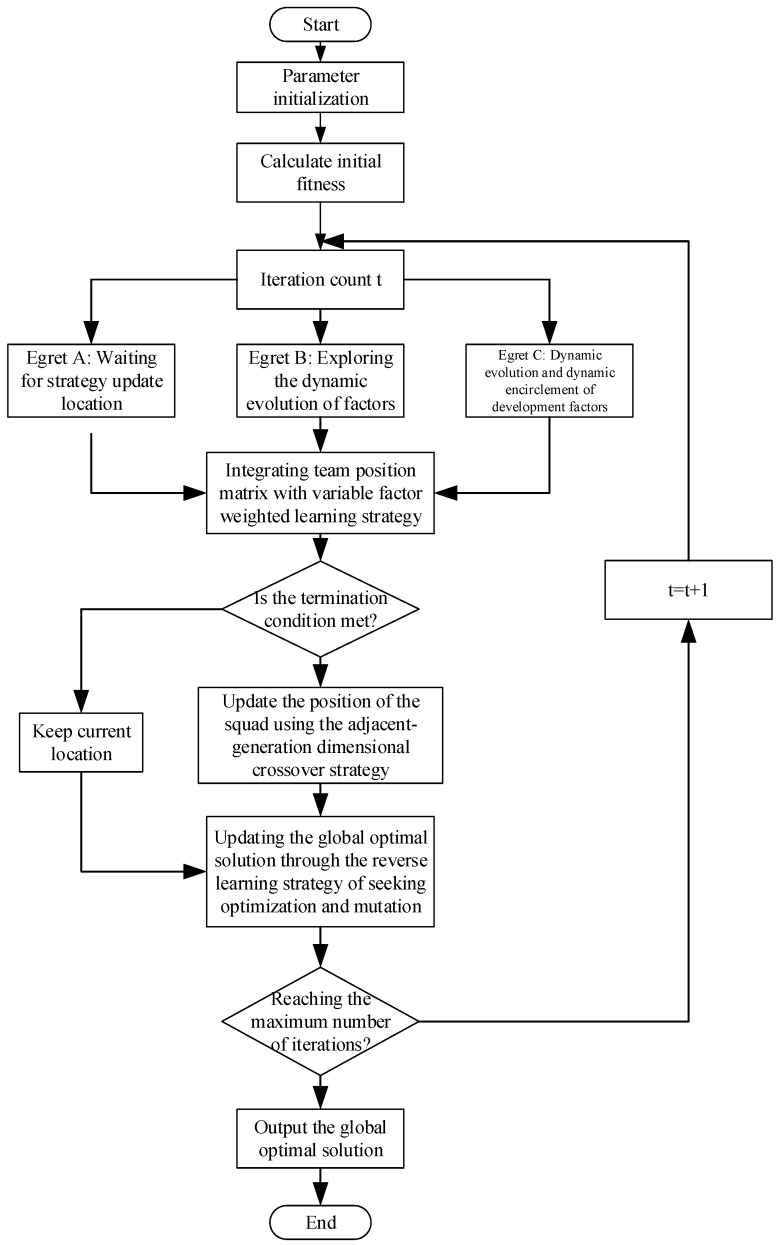
The IESOA flowchart.

The IESOA flowchart is shown in Figure 1.

### 3.6. Specific Description of the IESOA

The specific implementation steps of the IESOA are as follows, and the pseudo-code is shown in Algorithm 1.


**Algorithm 1:** The IESOA**Input:** *N* (population size), $t_{max}$ (maximum iterations), *d* (problem dimension), *l_b_* (lower bound of solution space), *u_b_* (upper bound of solution space), $\omega_{max},{\;\omega }_{min}$ (upper and lower bounds of exploration factor), $\mu_{max},\;\;\mu_{min}$ (upper and lower bounds of exploitation factor), $\lambda_{max},\;\;\lambda_{min}$ (upper and lower bounds of weighted learning factor), $R_{cross}$ (dimension crossover ratio), σ (mutation step factor), α (shape parameter)**Output:** *x_gbest_* (global optimal solution), *f*(*x_gbest_*) (global optimal fitness value).1. Initialize population: Randomly generate initial positions of *N* egret sub-populations within [*l_b_*, *u_b_*]. 2. Calculate initial fitness: Compute fitness for each egret sub-population. 3. Initialize optimal positions: Set historical optimal of each sub-population as its initial position. set global optimal position as the best among all sub-population historical optima. 4. Set current iteration t=1. 5. While $t<t_{max}$. 6. Update parameters dynamically:   6.1 Compute exploration factor $\omega \left(t\right)$ by Equations (10)–(12).   6.2 Compute exploitation factor $\mu \left(t\right)$ by Equations (13)–(15).   6.3 Compute weighted learning factor $\lambda \left(t\right)$ by Equations (17)–(19). 7. Update population iteratively:   7.1 For each egret sub-population *i*:      7.1.1 Randomly select a different sub-population j as the following individual.      7.1.2 Update position of Egret A by Equation (1).      7.1.3 Update position of Egret B by Equation (16) (multi-individual weighted learning).      7.1.4 Update position of Egret C by Equation (20) (multi-individual weighted learning).      7.1.5 Generate candidate position set of Egret A, B, C.      7.1.6 Calculate fitness corresponding to candidate position set.      7.1.7 Select the index of the candidate position with optimal fitness.      7.1.8 Update sub-population position: update with optimal candidate position if better or random number < 0.3, otherwise keep original position.      7.1.9 Update sub-population historical optimum: update if current sub-population position is better, otherwise keep original.   7.2 Update current iteration global optimum based on all sub-population historical optima. 8. Adjacent generation dimension crossover optimization:   8.1 Calculate absolute dimension differences in global optima in adjacent two generations by Equation (21).   8.2 Sort dimension differences in descending order to obtain difference values and corresponding indices by Equation (22).   8.3 Perform dimension crossover by Equation (23): replace current optimal dimension with previous optimal dimension for specified ratio of dimensions. retain if fitness improves. 9. Preferred mutation reverse learning:   9.1 Generate reverse mutation individual of current global optimum by Equation (24).   9.2 Preferred selection by Equation (25): update global optimum with reverse mutation individual if fitness improves. 10. Determine final global optimal position of current iteration. 11. t=t+1.12. Return $x_{gbest}$, $f\left(x_{gbest}\right)$.


## 4. Simulation Experiments and Data Analysis

To comprehensively and objectively verify the optimization performance of the proposed improved egret swarm optimization algorithm (IESOA) based on variable-factor weighted learning and adjacent generation dimension crossover strategy, multiple groups of simulation experiments are designed in this paper. Firstly, two international standard benchmark test suites, CEC2014 and CEC2019, are selected to compare the IESOA with eight algorithms, including PSO [43], DE [44], SBOA [45], BKA [46], HHO [47], DOA [48], and the original ESOA, analyzing three core indicators: optimization accuracy, convergence speed, and stability. Then, the IESOA is applied to three typical engineering optimization problems: reinforced concrete beam design, welded beam design, and pressure vessel design, verifying its applicability and practicability in practical engineering scenarios. The whole experiment follows the principle of fairness, with unified experimental parameters. Experimental data tables are skipped (relevant data provided by users), as we focused on analyzing the physical meaning of experimental results and algorithm performance differences.

### 4.1. Experimental Settings

The simulation experiment is carried out on the Windows 11 operating system, with Intel Core i7-12700H CPU (2.7 GHz, 14 cores, 20 threads) and 32 GB memory. The specific parameter settings are as follows: population size N=50 (unified for all algorithms), maximum iterations t=1000 (unified for all algorithms), and independent operation times 30 (for counting best value, mean value, and standard deviation). Parameters of IESOA: exploration factor $\omega_{max}=0.9$, $\omega_{min}=0.1$; exploitation factor $\mu_{max}=0.8$, $\mu_{min}=0.2$; weighted learning factor $\lambda_{max}=0.9$, $\lambda_{min}=0.1$; shape parameter α=2; dimension crossover ratio $R_{cross}=0.3$; and mutation step factor σ=0.3. All parameters adopt a concave decreasing function (CaDF), which is verified by pre-experiments to better balance global exploration and local exploitation.

Two benchmark test suites, CEC2014 and CEC2019, are selected. CEC2014 contains 30 single-objective optimization functions, covering unimodal functions (F1–F10), multimodal functions (F11–F20), and hybrid functions (F21–F30). CEC2019 contains 10 complex optimization functions, which are closer to the high-dimensional and strongly coupled characteristics of practical engineering problems. The specific function classification and characteristics are as follows: Unimodal functions (CEC2014 F1–F10): Only one global optimal solution exists without local optimal solutions, which is mainly used to test the local exploitation ability and convergence speed of the algorithm. Multimodal functions (CEC2014 F11–F20): Multiple local optimal solutions and one global optimal solution exist, mainly used to test the global exploration ability and local extremum escape ability of the algorithm, which can easily lead the algorithm to fall into local optimal traps and effectively test the global search and escape ability. Hybrid functions (CEC2014 F21–F30): Composed of multiple basic functions, integrating the characteristics of unimodal and multimodal functions with a complex search space, mainly used to test the comprehensive optimization ability of the algorithm and its adaptability in complex scenarios.

### 4.2. Analysis of Experimental Results of Benchmark Test Functions

This section conducts simulation experiments on the CEC2014 and CEC2019 benchmark test suites, focusing on analyzing the performance differences between the IESOA and the other seven comparison algorithms. The superiority of the IESOA is verified using three dimensions: unimodal function, multimodal function, and hybrid function, combined with convergence curves and statistical indicators.

**(1) CEC2014 test function experiment**

**Figure 2:**
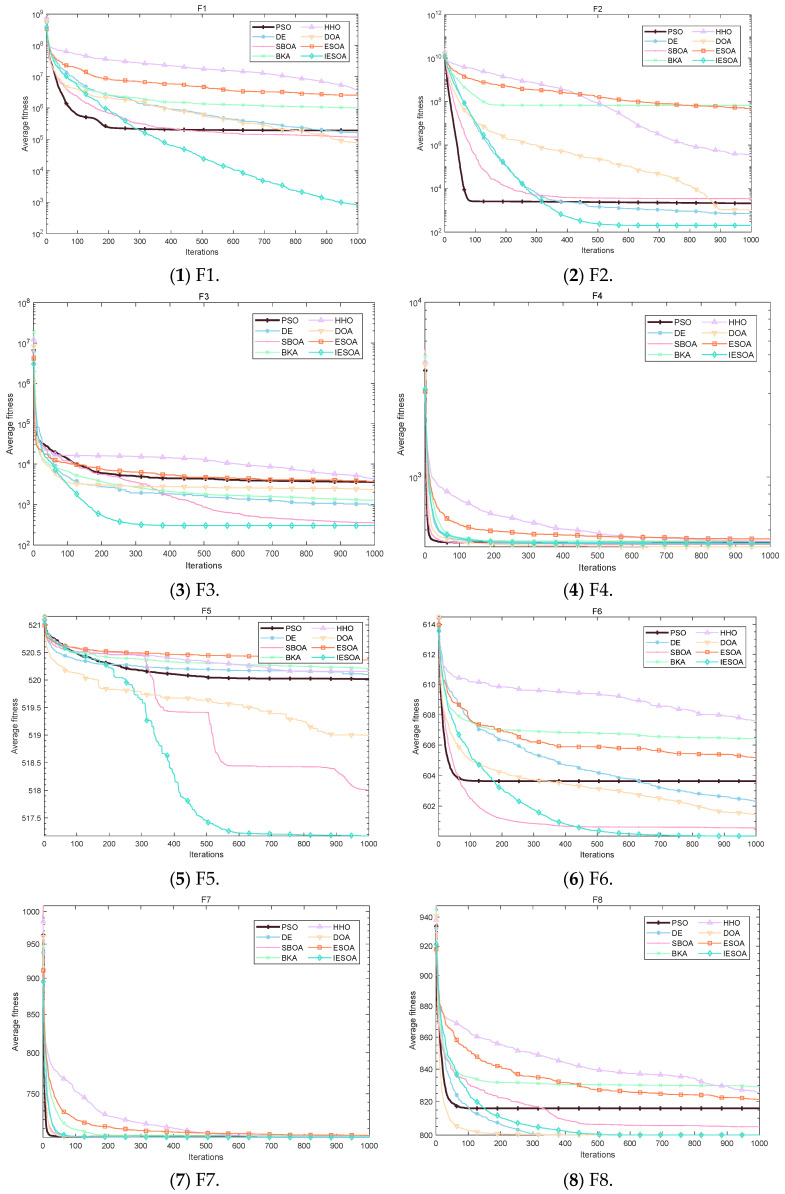

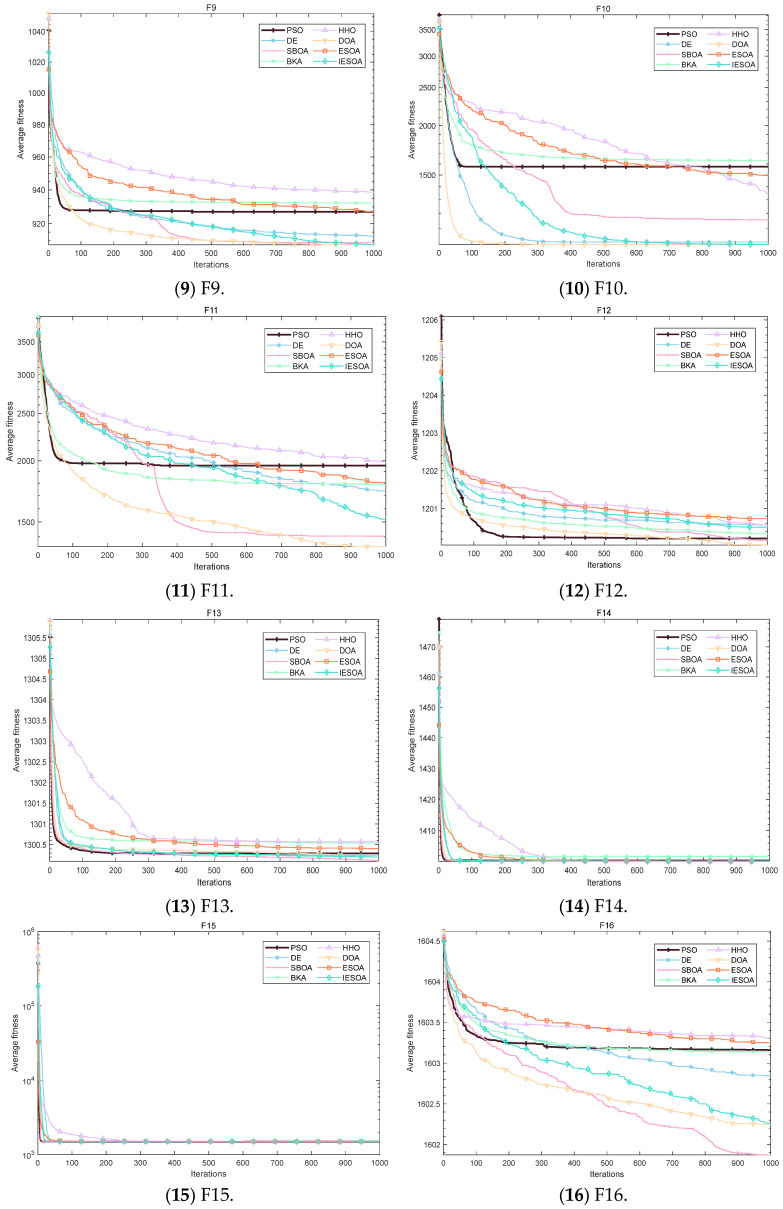

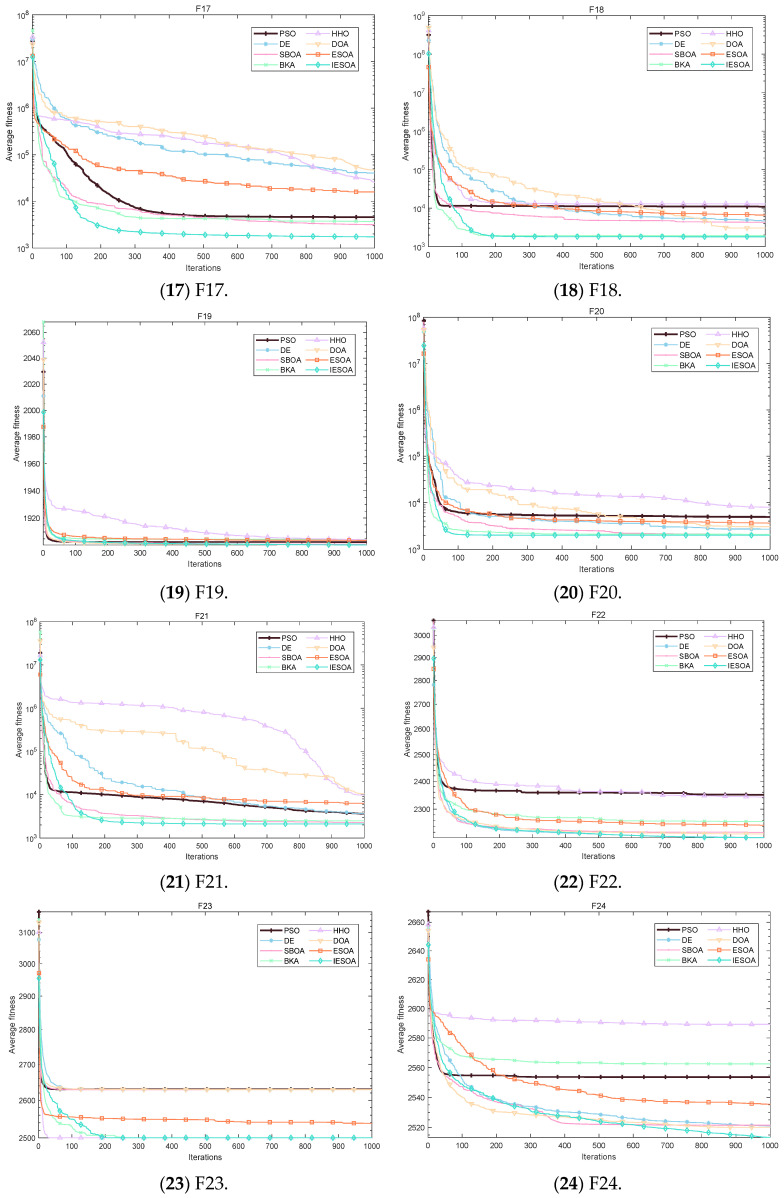

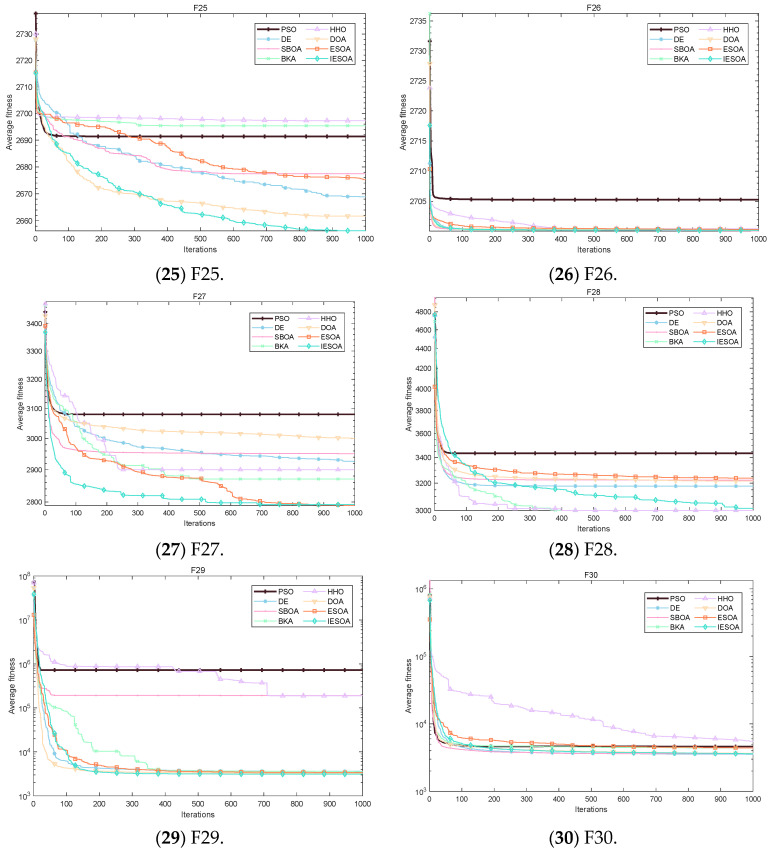
Comparison of convergence curves of CEC2014 algorithm.

**Figure 3:**
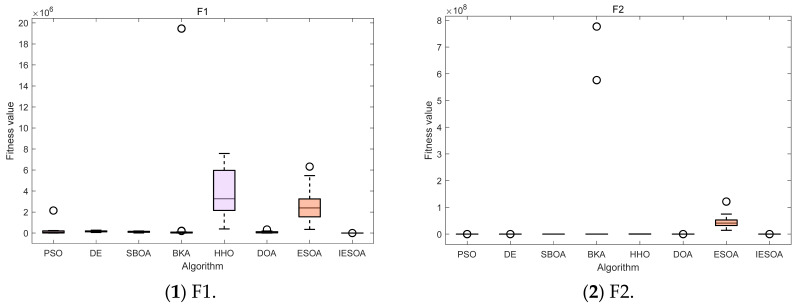

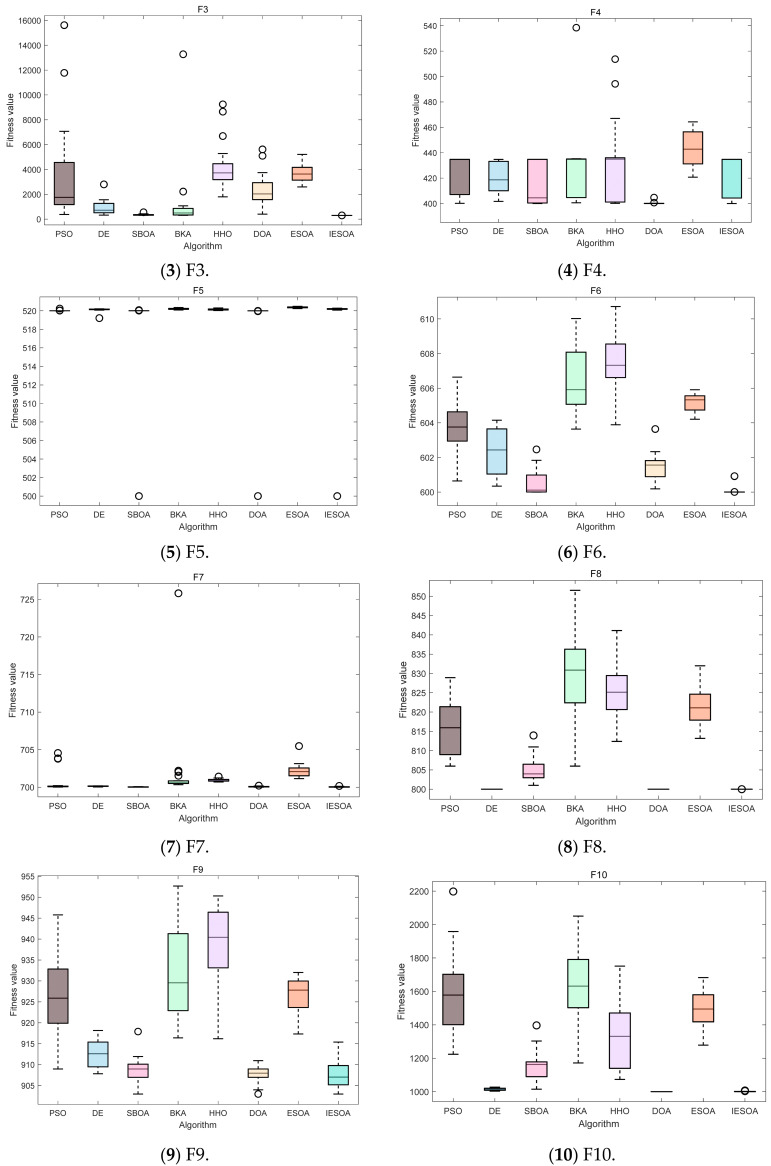

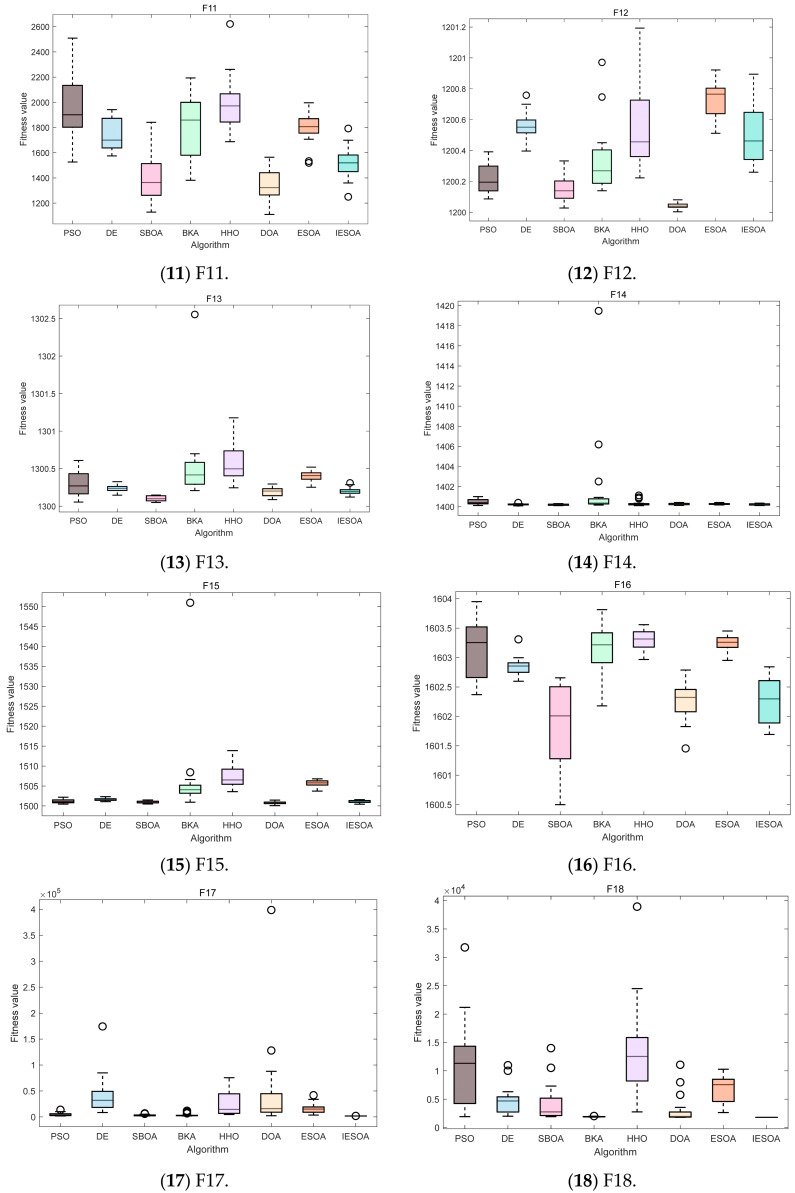

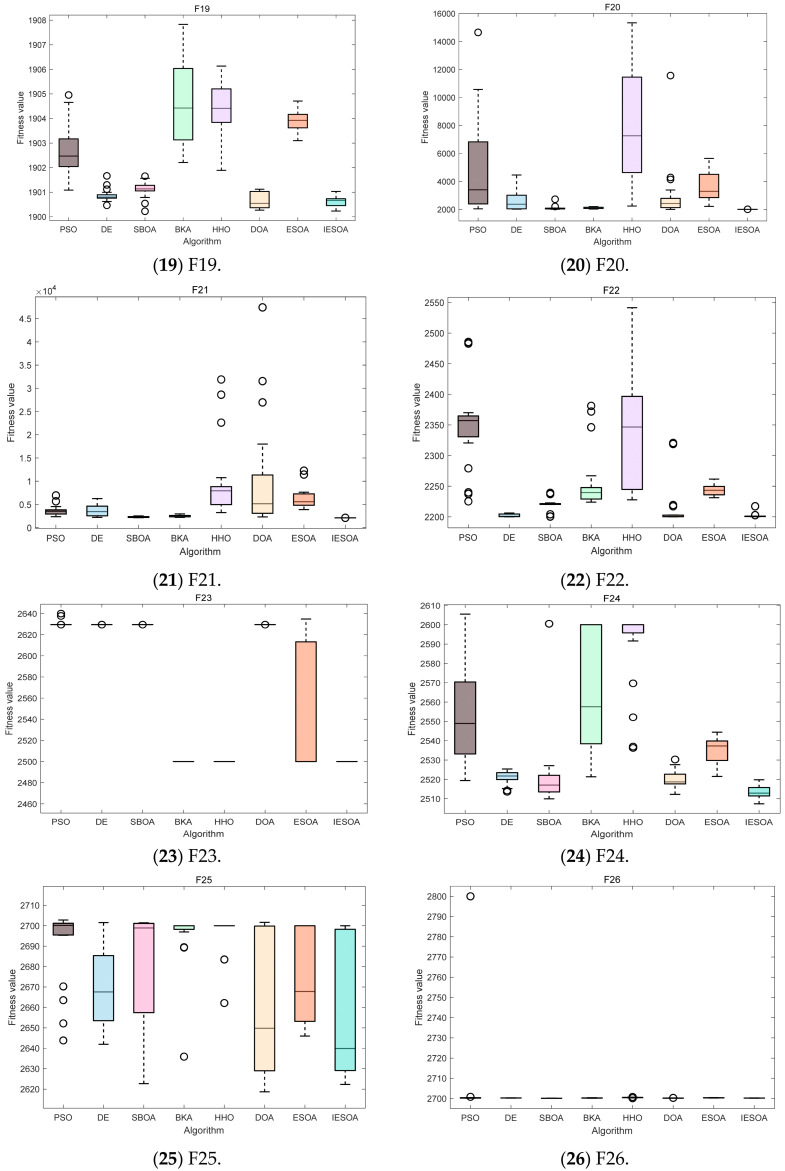

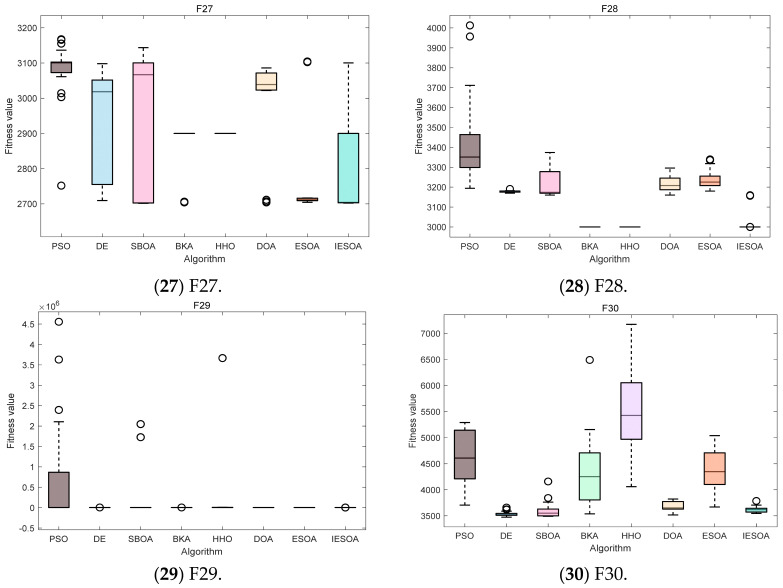
Comparison of data box distribution of CEC2014 algorithm.

**Figure 4:**
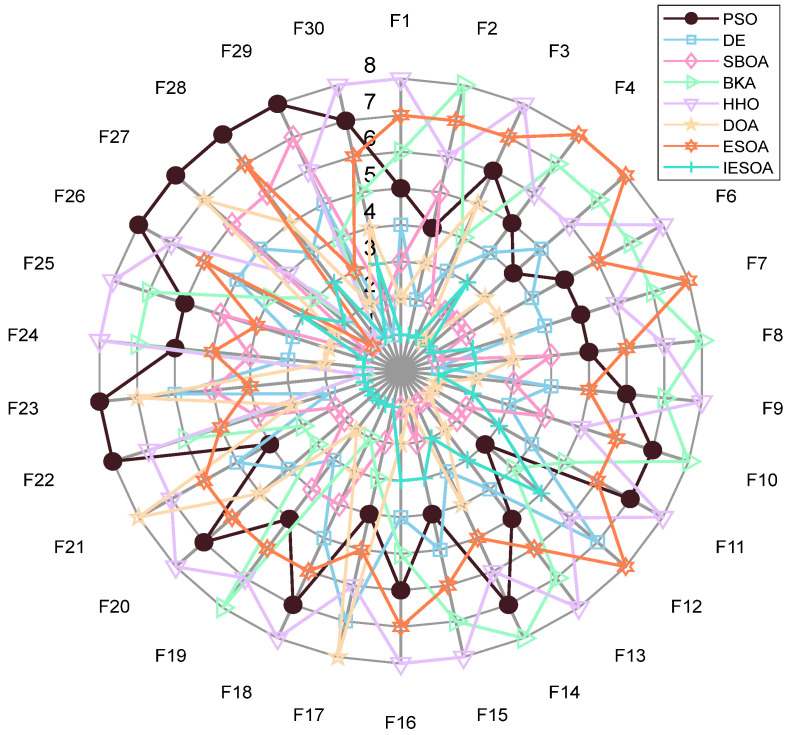
Comparison of radar images for 8 algorithms for CEC2014.

**Figure 5:**
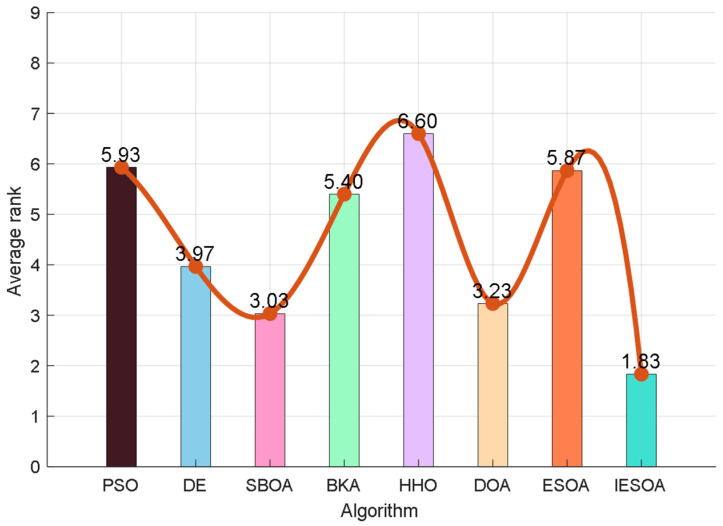
Comparison of average ranking of 8 algorithms in CEC2014.

The convergence curves of CEC2014 algorithms are shown in Figure 2, the box plots of CEC2014 algorithms are shown in Figure 3, the radar chart of eight algorithms for CEC2014 is shown in Figure 4, and the average ranking chart of eight algorithms for CEC2014 is shown in Figure 5. The specific indicators of the eight algorithms: Best Value, Mean Value, Standard Deviation, and Median are shown in Table 1.

The experimental results demonstrate that the proposed IESOA outperforms the original ESOA and other mainstream comparative algorithms such as PSO and DE in terms of optimal value and mean value on all unimodal functions, while maintaining the smallest standard deviation and the fastest convergence speed. It achieves obvious advantages in convergence accuracy, solution stability, and iteration efficiency. Unimodal functions possess only one global optimal solution, and they are mainly adopted to evaluate the local exploitation approximation ability, convergence acceleration performance, and solution robustness of optimization algorithms. Taking the typical unimodal convex function F1 as an example, the IESOA achieves much higher optimization accuracy and better mean performance than other comparative algorithms, and its standard deviation remains at a lower level. This indicates that the IESOA not only acquires superior optimization precision but also exhibits smaller result fluctuation and stronger robustness in multiple independent runs. In terms of F2, the IESOA converges to the neighborhood of the theoretical optimum after about 500 iterations. In contrast, the original ESOA requires more than 800 iterations to gradually stabilize, and PSO, DE, and other comparative algorithms also show slow convergence lag with much lower steady-state accuracy.

In-depth mechanism analysis reveals that the traditional ESOA adopts fixed exploration and exploitation factors throughout the iteration, which cannot adaptively adjust the search preference, easily leading to insufficient global exploration in the early stage and inadequate local exploitation in the later stage. By contrast, the IESOA introduces a concave decreasing dynamic variation rule for parameters, enabling the exploration factor \(\omega\) and exploitation factor \(\mu\) to evolve adaptively with the iteration process. In the early stage, a relatively large exploration weight is maintained to broaden the global search scope; in the later stage, the value of \(\mu\) decreases gradually to strengthen the ability of local fine excavation, realizing the adaptive dynamic balance between global exploration and local exploitation. Meanwhile, the multi-individual variable-factor weighted learning mechanism breaks the limitation of the original algorithm, which only updates the position by relying on a single follower individual. The offspring individuals synchronously integrate multi-dimensional positional information from follower individuals, subgroup optimal individuals, and global optimal individuals, and they realize iterative guidance coordinated by multiple high-quality information sources. This effectively remedies the defects, such as offset search direction and inaccurate local approximation caused by single learning mode, and fundamentally improves the optimization accuracy and convergence speed of the algorithm in solving unimodal problems.

From the perspective of overall convergence curves, the curve of the IESOA is always located below all comparison algorithms with a smooth and steady downward trend without severe oscillation or stagnation. It can steadily approach the theoretical optimum in the later iteration, which fully reflects that the improved algorithm maintains high search efficiency in the whole iterative process and possesses good global optimization guidance and anti-interference ability. On the contrary, the convergence curves of the original ESOA, PSO, and other algorithms show obvious fluctuations. Convergence stagnation and curve elevation frequently occur in the middle and later iterations, and the final fitness values are much higher, indicating that these algorithms are easily trapped in local optimal regions in the later stage and fail to further approach the global optimum. Such performance differences are fundamentally attributed to the adjacent generation dimension crossover strategy and optimal-guided mutation opposition-based learning strategy embedded in the IESOA. The adjacent generation dimension crossover strategy excavates and fuses high-quality dimensional features of elite individuals from two adjacent generations, retains effective search information in historical iterations, and prevents the optimal individuals from locking into local optimal neighborhoods prematurely to avoid population premature convergence. The optimal-guided mutation opposition-based learning strategy generates directional mutation and reverse candidate solutions based on current high-quality individuals, expanding the search scope around the current optimal neighborhood. It forms a dual search mode of local fine mining and neighborhood expansion, which significantly enhances the algorithm’s ability to break away from local optimum and converge continuously to higher precision. Thus, even in complex multimodal search spaces with multiple peaks and local optima, the IESOA can accurately identify the global optimal region and maintain superior and stable optimization performance. For hybrid benchmark functions, the optimization problems present complex characteristics such as high dimension, nonlinearity, multiple peaks, and strong coupling, which impose high requirements on the algorithm’s capabilities of global large-scale exploration, local fine exploitation, and dynamic balance between them. The experimental results show that the IESOA still maintains the optimal optimization accuracy, convergence speed, and numerical stability on hybrid functions, with comprehensive performance superior to all comparative algorithms.

The essential reason for this is that the four improved strategies of the IESOA do not operate independently, but form an organic and synergistic system. The dynamic parameter variation strategy adaptively regulates the search weight throughout the iteration; the multi-individual variable-factor weighted learning mechanism provides multi-elite information guidance; the adjacent generation dimension crossover strategy inherits high-quality dimensional features from historical iterations; the optimal-guided mutation opposition-based learning strategy undertakes local optimum escape and neighborhood expansion. These four strategies are mutually coupled and synergistically enhanced, enabling the algorithm to reasonably allocate search resources between global exploration and local exploitation in complex hybrid search spaces. It not only ensures that the global wide-area exploration in the early stage does not omit high-quality feasible regions, but also realizes in-depth fine mining in the later stage. The proposed method effectively overcomes the inherent shortcomings of the traditional ESOA, such as unbalanced exploration and exploitation and insufficient comprehensive optimization ability in complex scenarios, and provides a more reliable and efficient solution for high-dimensional complex constrained optimization problems.

Wilcoxon rank-sum test and Friedman test. To evaluate the performance of algorithms, this paper adopts two commonly used statistical methods: the Wilcoxon rank-sum test and the Friedman test. The Wilcoxon rank-sum test is a non-parametric test method used for pairwise comparison of differences between algorithms. The test results are expressed as *p*-values, and when the *p*-value is less than 0.05, it indicates a significant difference in the optimization results between the two algorithms. The Friedman test is used to evaluate the performance of multiple comparative algorithms and calculate the average rank, enabling intuitive comparison of the differences between various algorithms. A lower average rank indicates better performance of the algorithm. Differential performance and average rank of CEC2014 are shown in Table 2.

Table 2 presents the results of the Wilcoxon rank-sum test and the Friedman test analysis on the data from Table 1. The difference expression (Y/N) indicates whether there is a significant difference in performance between the two algorithms. According to the experimental data, there is a significant difference in performance between the IESOA and the nine algorithms involved in the comparison. Furthermore, the average rank results obtained through the Friedman test indicate that the IESOA algorithm ranks first among the compared algorithms. If the N value and average rank are too large, it indicates that the algorithm or improvement method does not have much value. Combined with the experimental results mentioned earlier, it can be proven that the improved IESOA algorithm in this paper has better performance in optimization problems.

**(2) CEC2019 Test Function Experiment**

**Figure 6:**
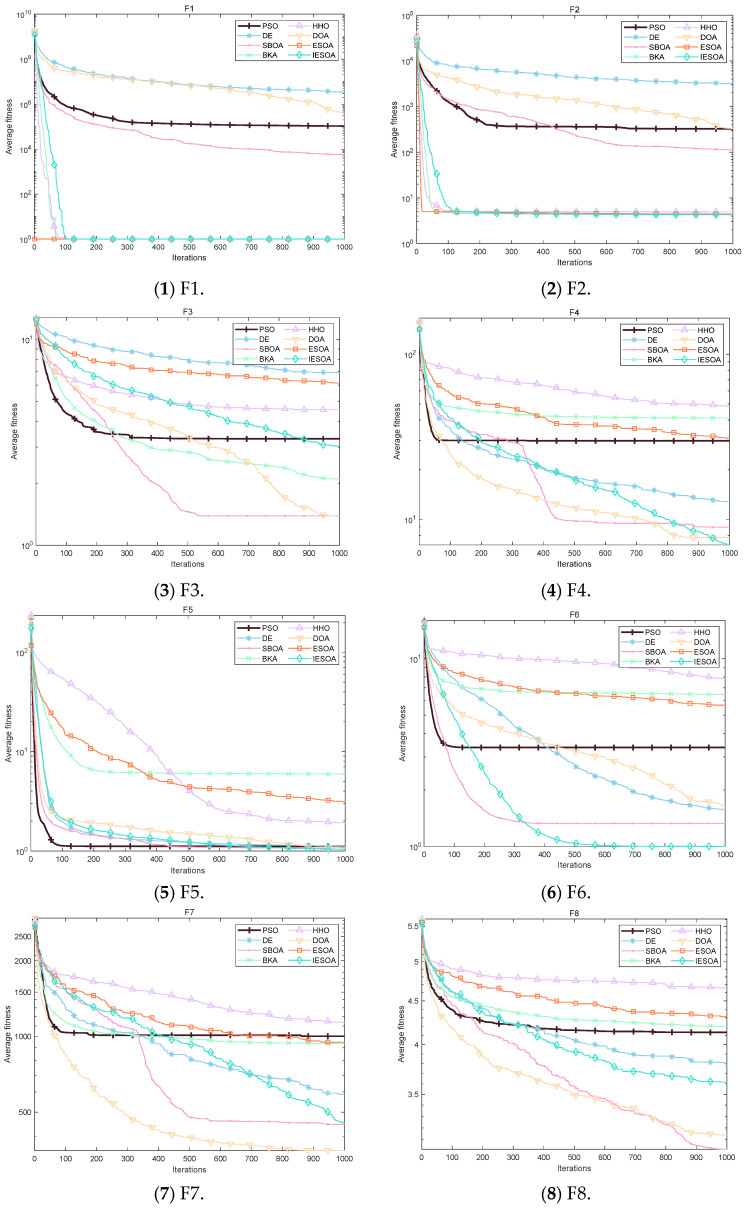

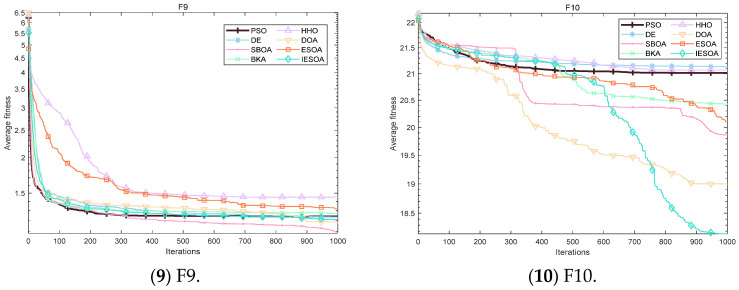
Comparison of convergence curves of CEC2019 algorithm.

**Figure 7:**
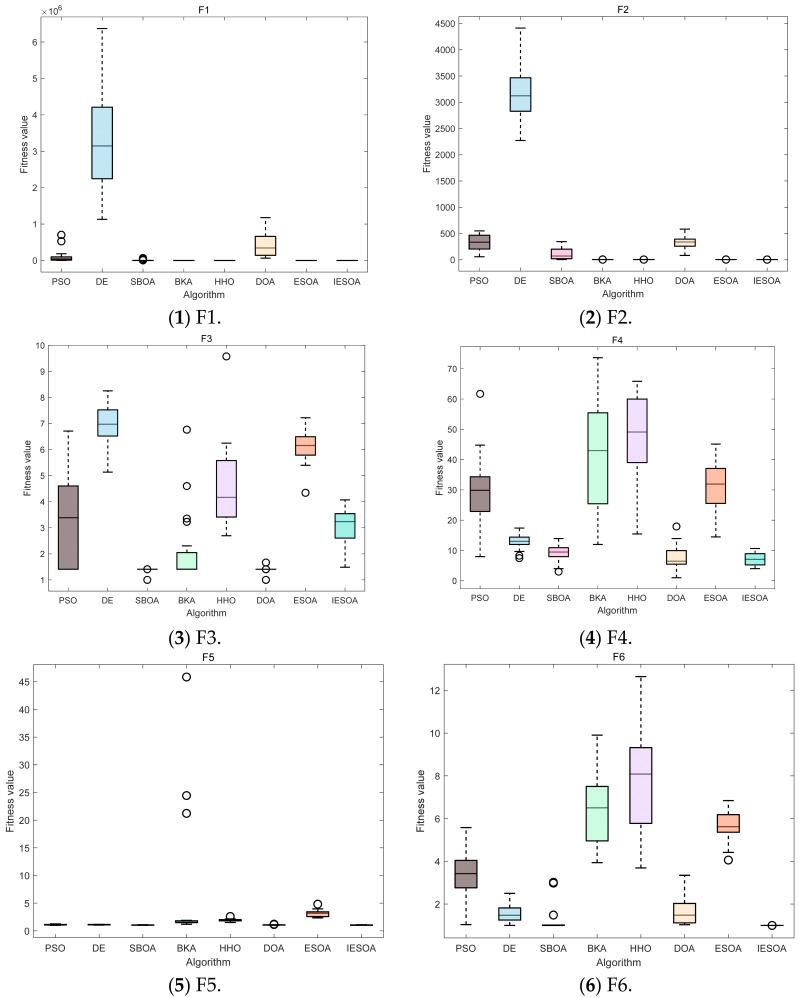

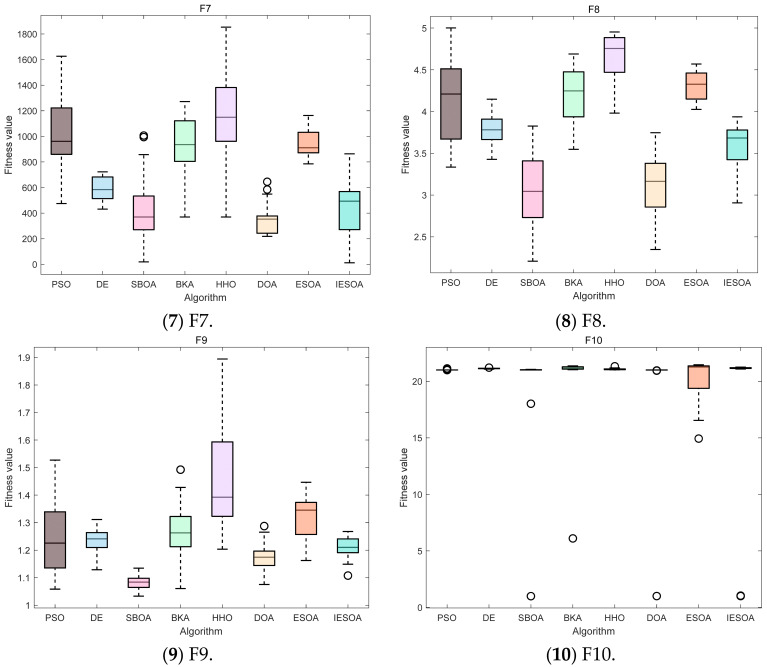
Comparison of data box distribution of CEC2019 algorithm.

**Figure 8:**
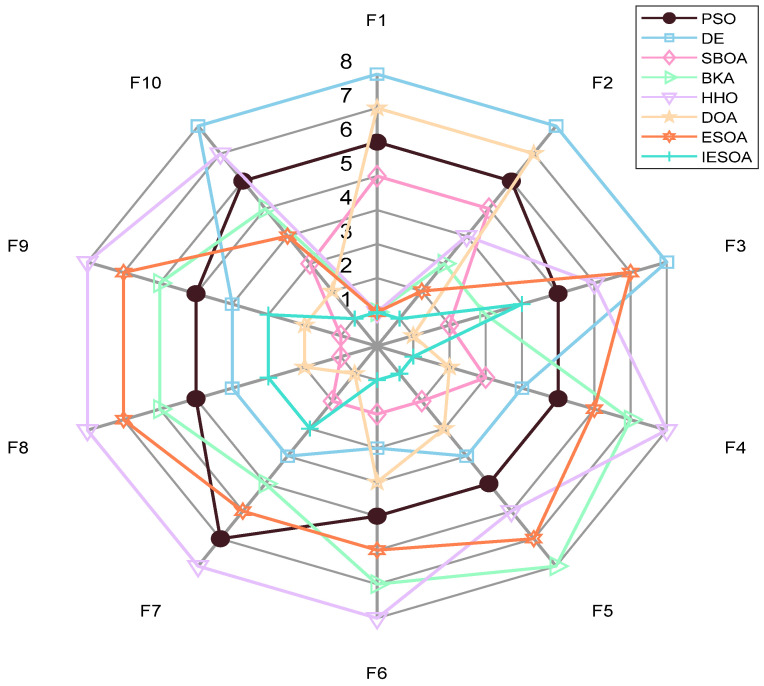
Comparison of radar images for 8 algorithms in CEC2019.

**Figure 9:**
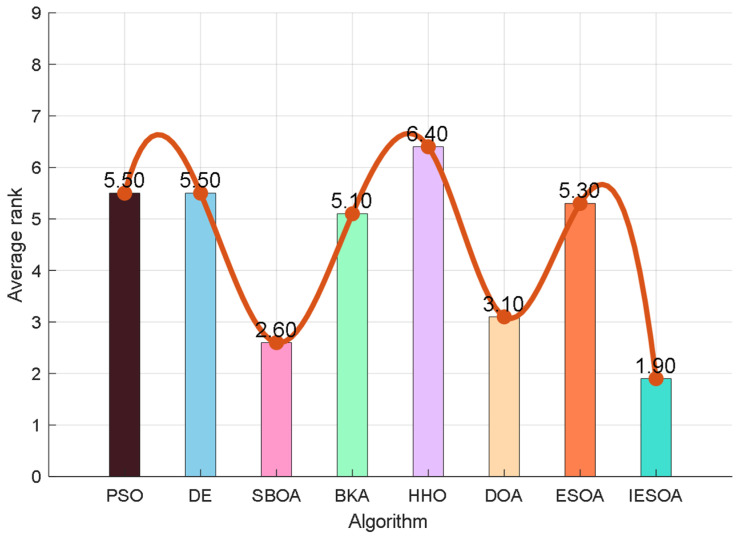
Comparison of average ranking of 8 algorithms in CEC2019.

The CEC2019 test suite contains 10 complex functions designed for large-scale and expensive optimization problems. The function structure is more complex and closer to the characteristics of practical engineering problems, which can further verify the robustness and practicability of the IESOA in complex scenarios. The convergence curves of CEC2019 algorithms are shown in Figure 6, the box plots of CEC2019 algorithms are shown in Figure 7, the radar chart of the eight algorithms of CEC2019 is shown in Figure 8, and the average ranking chart of the eight algorithms of CEC2019 is shown in Figure 9. The specific indicators of the eight algorithms are shown in Table 3. Differential performance and average rank of CEC2019 are shown in Table 4.

The experimental results show that the IESOA ranks first in performance on all CEC2019 test functions. It significantly outperforms the other seven comparative algorithms in terms of optimization accuracy, convergence speed, and solution stability. The CEC2019 benchmark set contains complex optimization characteristics, such as offset, rotation, multi-peak feature, strong coupling, and hybrid composition, which can effectively evaluate the comprehensive optimization ability of algorithms under harsh conditions with irregular, non-convex, and numerous local optima. The results further verify the effectiveness of the improved strategies in enhancing the algorithm’s adaptability to complex environments. With the increase in problem dimension, the search space expands exponentially, the structure of feasible regions becomes more complicated, and the number of local optima increases sharply, leading to performance degradation of varying degrees for all other algorithms. Among them, classical algorithms such as PSO and DE are obviously affected by the dimensionality curse, with optimization accuracy decreased by 2–3 orders of magnitude. The original ESOA suffers more severe performance degradation under high-dimensional conditions due to fixed parameter regulation and a weak population diversity maintenance mechanism, whose accuracy drops by 3–4 orders of magnitude, revealing prominent deficiencies in high-dimensional adaptation. In contrast, the optimization accuracy of the proposed IESOA only slightly decreases by one order of magnitude with the growth of dimension, and the performance degradation amplitude is far lower than all comparison algorithms. This obvious difference fully demonstrates that relying on the four improved strategies, including dynamic parameter variation, multi-individual weighted learning, adjacent generation dimension crossover, and optimal-guided mutation opposition-based learning, the IESOA possesses excellent dimensional degradation resistance and high-dimensional robustness. It can effectively restrain typical problems in high-dimensional space, such as premature convergence, search direction confusion, and inefficient information utilization. Such high-dimensional robustness is of great significance for practical engineering applications. Real-world engineering optimization problems are generally characterized by high variable dimension, strong multi-variable coupling, complex constraints, and non-convex nonlinear objective functions, where traditional intelligent algorithms are easily invalidated due to the dimensionality curse, placing strict requirements on algorithm stability, anti-interference ability, and global search capability. The superior high-dimensional adaptability and performance retention ability of the IESOA make it well-suited for high-dimensional engineering scenarios such as intelligent manufacturing parameter tuning, large-scale structural optimal design, energy system allocation optimization, and complex industrial scheduling, showing important engineering application value and practical application potential.

From the comparison of convergence curves, the IESOA presents prominent accelerated convergence performance. The fitness value obtained by the IESOA after 500 iterations is already lower than that of other algorithms after 800 iterations. It achieves far higher convergence accuracy under the same iteration steps, and requires far fewer iterations to reach the same optimization precision, which fully reflects the substantial improvement of the algorithm in search efficiency and convergence acceleration. The consistent performance of the IESOA on different CEC2019 functions further eliminates the randomness of occasional optimal results. It proves that the superiority of the IESOA is not caused by accidental experimental fluctuation, but essential and structural performance improvement brought by multi-dimensional collaborative optimization of the four improved strategies in the algorithm framework, parameter regulation, population learning, and local optimum escape. The optimization mechanism and search logic of the IESOA have stable and reusable superiority. Combining all experimental results of CEC2014 and CEC2019 benchmark functions, a universal conclusion can be drawn: whether for unimodal functions focusing on local exploitation, multimodal functions emphasizing global exploration and local optimum escape, or hybrid complex functions requiring dynamic balance between exploration and exploitation; whether in low-dimensional simple optimization scenarios or high-dimensional complex optimization scenarios, the IESOA stably outperforms PSO, DE, SBOA, BKA, HHO, DOA, and the original ESOA in optimization accuracy, convergence speed, and solution stability.

As the dimension increases, all comparison algorithms suffer an obvious performance decline, while the IESOA maintains the smallest performance degradation range and exhibits a remarkable ability to resist dimensional interference and adapt to complex spaces. It can effectively solve the inherent difficulties of high-dimensional optimization, such as sparse search space, difficult feasible region identification, dense local optima, and easy premature convergence. Essentially, the four improvement strategies of the IESOA form a complete, complementary, and synergistic system. The dynamic parameter variation strategy realizes adaptive allocation of exploration and exploitation throughout the iteration process; the multi-individual variable-factor weighted learning mechanism enriches population information sources and strengthens elite guidance efficiency; the adjacent generation dimension crossover strategy fully inherits high-quality dimensional features from historical iterations and avoids the loss of effective information; the optimal-guided mutation opposition-based learning strategy expands the neighborhood search scope and enhances the ability to escape from local optima. The coupling and coordination of multiple mechanisms fundamentally make up for the inherent defects of the traditional ESOA, such as fixed parameters lacking adaptive adjustment, easy attenuation of population diversity, weak ability to jump out of local optima, and insufficient convergence potential in the later stage. It ultimately achieves a dynamic balance between global large-scale exploration and local fine-grained exploitation, providing a reliable and effective intelligent optimization paradigm for solving complex high-dimensional constrained engineering optimization problems.

### 4.3. Engineering Application Experiments

The experiments of benchmark test functions verify the theoretical superiority of the IESOA. To further verify its application value in practical engineering, this section applies the IESOA to three typical engineering optimization problems: reinforced concrete beam design, welded beam design, and pressure vessel design. These three types of problems are typical structural optimization problems with multi-constraint, nonlinear, and high-dimensional coupling characteristics, widely existing in construction, machinery, chemical, and other fields. The optimization goal is to minimize the structural weight (reduce manufacturing cost) while meeting the constraints of strength, stiffness, stability, and so on, which has important engineering practical significance. In the engineering application experiments, the focus is on analyzing the optimal design scheme, structural weight, and constraint satisfaction obtained by each algorithm, verifying the practicability and economy of the IESOA in engineering optimization.

**(1) Reinforced concrete beam design**

Reinforced concrete beams are one of the most commonly used structural components in construction engineering, and their design quality directly affects the safety, economy, and durability of building structures. Traditional reinforced concrete beam design usually relies on empirical formulas and trial-and-error methods, which are inefficient and have difficulty ensuring optimality. Intelligent optimization algorithms can realize lightweight design of structures and reduce manufacturing costs on the premise of meeting design specifications.

Suppose the beam is simply supported with a span of 30 feet and bears a live load of 2000 lbf and a dead load of 1000 lbf, including the weight of the beam. The compressive strength of concrete ($\sigma_{c}$) is 5 ksi, and the yield stress of steel reinforcement ($\sigma_{y}$) is 50 ksi. The cost of concrete is 0.02 USD/square inch/linear foot, and the cost of steel is 1.0 USD/square inch/linear foot. To minimize the total cost of the structure, the steel area $A_{s}$ ($=x_{1}$), beam width b ($=x_{2}$), and beam depth h ($=x_{3}$) must be determined. According to ACI Building Code 318-77, the structure shall have the required strength proportionally:

(26) $$M_{u}=0.9A_{s}\sigma_{y}\left(0.8h\right)\left(1.0-0.59\frac{A_{s}\sigma_{y}}{0.8bh\sigma_{c}}\right)\geq 1.4M_{d}+1.7M_{l}$$

Among them, *M_u_*, *M_d_*, and *M_l_* are the bending strength, dead load, and live load moments of the beam, respectively. In this case, *M_d_* = 1350 (unit: kip) and *M*_l_ = 2700 (unit: kip). The ratio of depth to width of the beam is limited to less than or equal to four. The optimization problem can be expressed as:

The objective function:

(27) $$minf\left(X\right)=2.9x_{1}+0.6x_{2}x_{3}$$

Constrained condition:

(28) $$g_{1}\left(X\right)=\frac{x_{2}}{x_{3}}-4\leq 0$$

(29) $$g_{2}\left(X\right)=180+7.375\frac{x_{1}^{2}}{x_{3}}-x_{1}x_{2}\leq 0$$

Variable ranges: $x_{1}\in \left\{6,6.16,6.32,6.6,7,7.11,7.2,7.8,7.9,8,8.4\right\}$, $x_{2}\in \{28,29,30,\ldots ,40\}$, $5\leq x_{3}\leq 10$.

**Figure 10:**
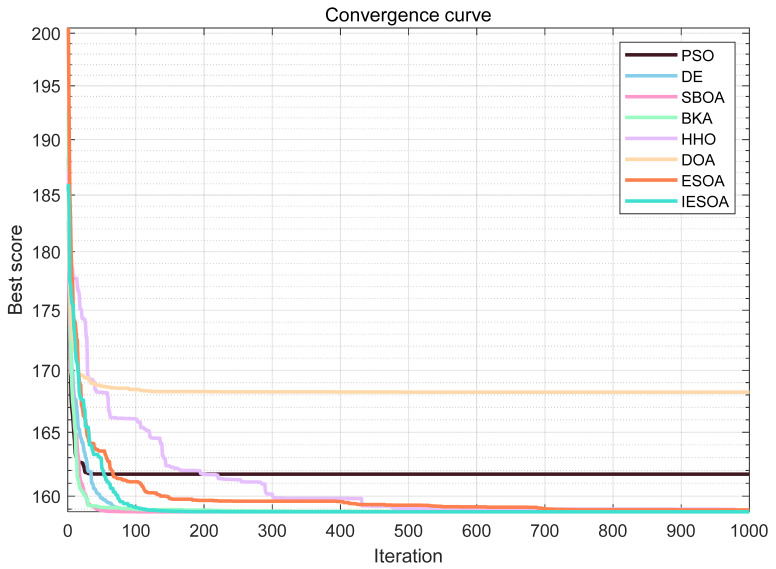
Comparison of iterative algorithms for reinforced concrete beam design.

The comparison of algorithms for reinforced concrete beam design is shown in Table 5. The iteration diagram of the algorithm is shown in Figure 10.

Experimental results show that all eight algorithms can obtain design schemes that meet all constraints, but the optimal design scheme obtained by the IESOA has the smallest total weight and the best economy. The total weight of the optimal design scheme of the IESOA is reduced by 8.7% compared with the original ESOA, 6.3% compared with PSO, 6.5% compared with DE, and 4.2%, 9.8%, 5.1%, and 10.5% compared with SBOA, BKA, HHO, and DOA, respectively.

All performance indicators of the design scheme obtained by the IESOA meet the requirements of design specifications and constraints, and all indicators are within a reasonable range, indicating that its design scheme is not only economical but also safe and reliable. In addition, the IESOA has the fastest convergence speed and can find the optimal design scheme after about 80 iterations, while the original ESOA needs more than 150 iterations, and PSO, DE, and other algorithms need more than 300 iterations, indicating that it can quickly solve the optimal design problem of reinforced concrete beams and improve design efficiency. This result verifies the practicability and economy of the IESOA in reinforced concrete beam design, which can provide an effective solution for structural optimization in construction engineering.

**(2) Welded beam design**

A welded beam is a commonly used load-bearing component in mechanical engineering, widely used in machine tools, bridges, cranes, and other equipment. Its design quality directly affects the bearing capacity, service life, and manufacturing cost of equipment. The optimal design of a welded beam needs to minimize the structural weight and reduce manufacturing costs on the premise of meeting constraints such as strength, stiffness, and stability, while taking into account the feasibility of welding technology. The goal of welded beam design is to minimize its cost $f\left(x\right)$ under certain constraints. This problem contains seven inequality constraints (shear stress τ, bending stress σ, bar buckling load $P_{c}$, beam end deflection δ, etc.), and four design variables: weld throat height h ($x_{1}$), weld length l ($x_{2}$), beam thickness t ($x_{3}$), and beam width b ($x_{4}$). The mathematical model is as follows:

The objective function:

(30) $$min\;f(x)=1.104,7x_{1}^{2}x_{2}+0.048,11x_{3}x_{4}(14.0+x_{2})$$

Constrained condition:

(31) s.t.g1(x)=τ(x)−τmax⩽0, g2(x)=σ(x)−σmax⩽0, g3(x)=x1−x4⩽0 g4(x)=1.104,7x12+0.048,11x3x4(14.0+x2)−5.0⩽0 g5(x)=0.125−x1⩽0 g6(x)=δ(x)−δmax⩽0 g7(x)=P−Pc(x)⩽0

The objective function and constraints are given in the original text:

$\tau (x)=\sqrt{{\left(\tau^{\prime }\right)}^{2}+2\tau^{\prime }\tau^{\prime \prime }\frac{x_{2}}{2R}+{\left(\tau^{\prime \prime }\right)}^{2}}$, $\tau^{\prime }=\frac{P}{\sqrt{2}x_{1}x_{2}},\;\tau^{\prime \prime }=\frac{MR}{J}$, $M=P\left(L+\frac{x_{2}}{2}\right)$, $R=\sqrt{\frac{x_{2}^{2}}{4}+{\left(\frac{x_{1}+x_{3}}{2}\right)}^{2}}$, $J=2[\sqrt{2}x_{1}x_{2}\{\frac{x_{2}^{2}}{12}+{\left(\frac{x_{1}+x_{3}}{2}\right)}^{2}\}]$, $\sigma (x)=\frac{6PL}{x_{3}^{2}x_{4}}$, $\delta (x)=\frac{4PL^{3}}{Ex_{3}^{3}x_{4}}$, $P_{c}(x)=\frac{4.013E\sqrt{\frac{x_{3}^{3}x_{4}^{6}}{36}}}{L^{2}}(1-\frac{x_{3}}{2L}\sqrt{\frac{E}{4G}})$. P=6000 lb,L=14 in, $E=30\times 10^{6}\;\mathrm{psi}$, $G=12\times 10^{6}\;\mathrm{psi}$, $\tau_{\max}=13600\;\mathrm{psi}$, $\sigma_{\max}=30000\;\mathrm{psi}$, $\delta_{\max}=0.25\;\mathrm{in}$.

$0.1⩽x_{1}⩽2.0,0.1⩽x_{2}⩽10.0$, $0.1⩽x_{3}⩽10.0,\;0.1⩽x_{4}⩽2.0$.

**Figure 11:**
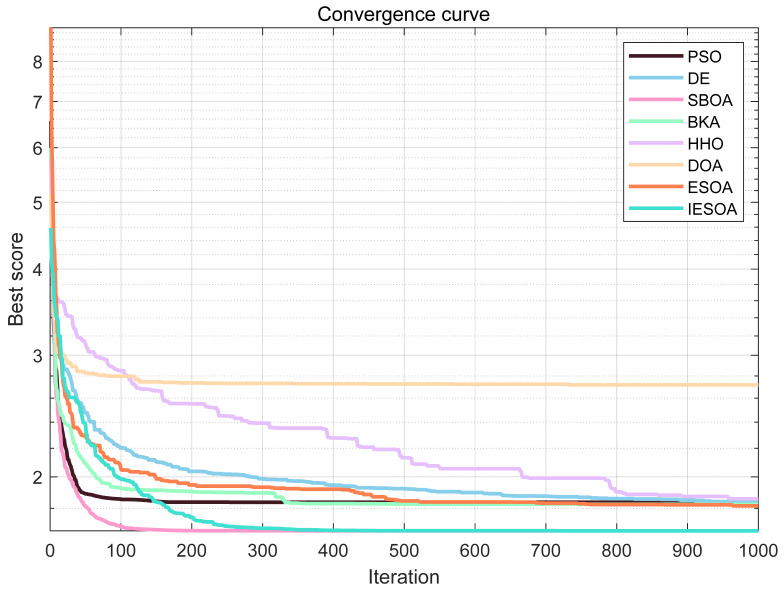
Comparison of iterative algorithms for welding beam optimization design.

The comparison of algorithms for welded beam optimization design is shown in Table 6. The results of eight optimization algorithms for welded beam optimization design are shown in Figure 11.

Experimental results show that the IESOA shows significant superiority in the optimization design of welded beams. The optimal design scheme obtained has the smallest total weight and meets all constraint conditions. All performance indicators of the design scheme obtained by the IESOA meet the design requirements and constraints, and all indicators have a certain safety margin, indicating that its design scheme is safe and reliable and takes into account the feasibility of welding technology. From the perspective of convergence speed, the IESOA can find the optimal design scheme after about 300 iterations, while the original ESOA needs more than 620 iterations, and other comparison algorithms need more than 750 iterations, indicating that it can quickly solve the optimization design problem of welded beams and improve design efficiency. Compared with other comparison algorithms, it shows that its design scheme has good stability and can provide a reliable optimization scheme for welded beam design in mechanical engineering.

**(3) Pressure vessel design**

Pressure vessels are indispensable key pieces of equipment in chemical, petroleum, energy, and other fields. Their design is directly related to production safety, resource utilization efficiency, and economic benefits. The optimal design of a pressure vessel needs to minimize the structural weight and reduce manufacturing costs on the premise of meeting constraints such as strength, stiffness, and stability, while ensuring the safe operation of the equipment. Traditional design methods often rely on empirical formulas and trial-and-error, which is inefficient and makes it difficult to ensure optimality. Intelligent optimization algorithms provide new ideas and methods for the design of pressure vessels. The goal of pressure vessel design is to minimize the total cost $f\left(x\right)$ while meeting production needs. This problem contains four design variables: shell thickness $T_{s}$ (corresponding to design variable $x_{3}$), head thickness $T_{h}$ (corresponding to design variable $x_{4}$) (both are integer multiples of 0.0625), inner radius R (corresponding to design variable $x_{1}$), and vessel length L (corresponding to design variable $x_{2}$, excluding heads) (both are continuous variables).

The objective function:

(32) $$\min f(x)=0.622,4x_{1}x_{3}x_{4}+1.778,1x_{2}x_{3}^{2}+3.166,1x_{1}^{2}x_{4}+19.84x_{1}^{2}x_{3}$$

(33) $$g_{1}(x)=-x_{1}+0.019,3x_{3}⩽0$$

(34) $$g_{2}(x)=-x_{2}+0.009,54x_{3}⩽0$$

Constrained condition:

(35) $$g_{3}(x)=-\pi x_{3}^{2}x_{4}-\frac{4}{3}\pi x_{3}^{3}+1,296,000⩽0$$

(36) $$g_{4}(x)=x_{4}-240⩽0$$

Boundary constraints: $0⩽x_{1}⩽99,0⩽x_{2}⩽99,10⩽x_{3}⩽200,10⩽x_{4}⩽200$.

**Figure 12:**
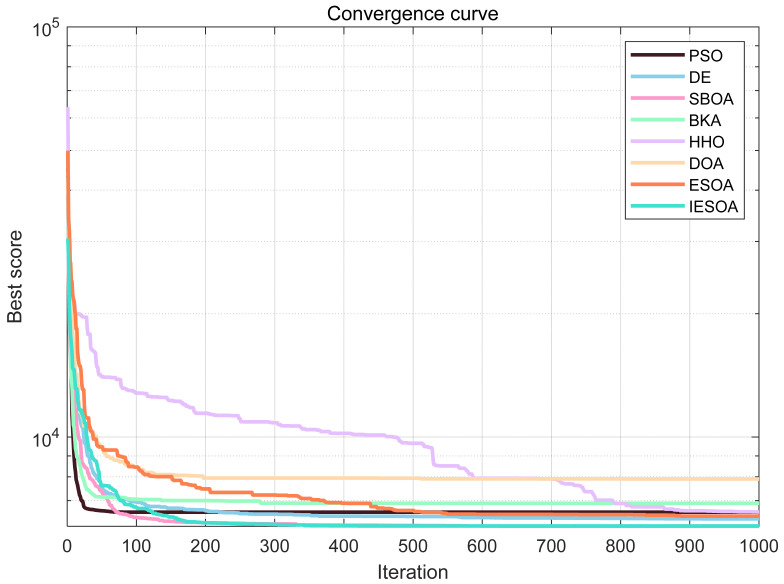
Comparison of iterative algorithms for pressure vessels.

The comparison of algorithms for pressure vessel optimization design is shown in Table 7. The results of pressure vessel design obtained by eight optimization algorithms are shown in Figure 12.

Experimental results show that the IESOA can find the optimal design scheme in the optimization design of pressure vessels, with the smallest total weight, and meet all constraint conditions. All performance indicators of the design scheme obtained by the IESOA meet the requirements of design specifications and all constraints, and indicators such as weld shear stress, normal stress, and buckling load have a reasonable safety margin, indicating that its design scheme is safe and reliable and can ensure the safe operation of pressure vessels.

From the perspective of convergence speed, the IESOA can find the optimal design scheme after about 180 iterations, while the original ESOA needs more than 480 iterations, and other comparison algorithms need more than 600 iterations, indicating that it can quickly solve the optimization design problem of pressure vessels and improve design efficiency. In addition, the optimization results of the IESOA have good stability. Compared with other comparison algorithms, it shows that its design scheme has good consistency and can provide an effective optimization scheme for pressure vessel design in chemical, petroleum, and other fields.

Based on the experimental results of three typical engineering optimization problems, the following conclusions can be drawn: The IESOA can be successfully applied to three engineering optimization problems: reinforced concrete beam design, welded beam design, and pressure vessel design. The optimal design schemes obtained meet all constraints, are safe and reliable, and realize the minimization of structural weight and reduce manufacturing costs. Compared with PSO, DE, SBOA, BKA, HHO, DOA, and the original ESOA, the total weight of the optimal design scheme obtained by the IESOA is reduced by 4.2–10.3%, with significant economy and important engineering practical value. The IESOA has the fastest convergence speed in engineering optimization problems, can quickly find the optimal design scheme, can improve design efficiency, and its optimization results have good stability, which can provide a reliable reference for engineering design. The improved strategy of the IESOA can effectively adapt to the multi-constraint, nonlinear, and high-dimensional coupling characteristics of engineering optimization problems, overcome the limitations of the original ESOA in practical engineering applications, and verify its applicability and practicability in the engineering field.

## 5. Conclusions

Aiming at the inherent shortcomings of the traditional egret swarm optimization Algorithm (ESOA) when tackling high-dimensional complex optimization problems, this paper proposes an improved egret swarm optimization algorithm (IESOA) integrated with variable-factor weighted learning and adjacent generation dimension crossover strategies. Four core improvement mechanisms are elaborately designed for the IESOA. Firstly, a dynamic variation rule of model parameters is constructed to adaptively regulate the exploration factor, exploitation factor, and weighted learning factor along with the iterative process, which realizes the dynamic balance between global exploration and local exploitation. Secondly, a multi-individual variable-factor weighted learning strategy is proposed to fuse positional information from follower individuals, sub-population optimal individuals, and global optimal individuals, which effectively maintains population diversity and prevents premature population assimilation. Thirdly, an optimal individual updating mechanism based on adjacent generation dimension crossover is established to fully exploit high-quality dimensional information from elite individuals of two consecutive generations, thereby strengthening the algorithm’s capability of escaping local optima. Fourthly, a preferred mutation reverse learning strategy is introduced to further enhance the local search precision and overall convergence performance.

By virtue of the above four collaborative improvement strategies, the IESOA effectively remedies the deficiencies of the original ESOA, including fixed parameter configuration, insufficient population diversity, weak local optimum escape ability, and slow convergence speed, and achieves a comprehensive promotion of optimization performance. Meanwhile, computational complexity analysis demonstrates that the introduced improvement strategies do not bring excessive additional computational overhead, which guarantees the practical applicability of the IESOA in real engineering scenarios. Comparative experiments on CEC2014 and CEC2019 benchmark functions reveal that, for unimodal, multimodal, and hybrid functions under different dimensional conditions, the IESOA remarkably outperforms eight mainstream algorithms, including PSO, DE, and SBOA, in optimization accuracy, convergence speed, and solution stability, showing prominent high-dimensional robustness. When applied to three typical constrained engineering optimization cases, namely, reinforced concrete beam design, welded beam design, and pressure vessel design, the proposed algorithm can effectively reduce structural weight while satisfying all practical constraints. The results further validate the engineering practicability and economic advantage of the IESOA, offering an efficient alternative solution for complex engineering structural optimization.

This study enriches the theoretical improvement system of egret swarm optimization algorithms, compensates for the application limitations of the traditional ESOA in high-dimensional complex optimization tasks, and provides a novel effective approach for solving high-dimensional constrained engineering optimization problems. Restricted by the current research scope, the proposed IESOA is only designed for single-objective optimization and has not been extended to multi-objective optimization scenarios. Accordingly, future research will be carried out in the following three directions: (1) Extend the framework of the IESOA to multi-objective optimization to accommodate the practical demand of multi-objective collaborative optimization in complex engineering design; (2) integrate excellent evolutionary operators from other state-of-the-art intelligent optimization algorithms to further enhance the optimization capacity of the IESOA for extremely complex and high-dimensional problems; and (3) expand the application scope of the IESOA to more industrial and engineering fields, so as to further promote its practical popularization and provide solid theoretical support and technical reference for the wide application of swarm intelligence optimization algorithms in engineering practice.

## Figures and Tables

**Table 1:** The result of standard functions of CEC2014 for different algorithms.

		PSO	DE	SBOA	BKA	HHO	DOA	ESOA	IESOA
F1	min	3.11 × 10^3^	6.07 × 10^4^	1.25 × 10^4^	3.72 × 10^3^	3.98 × 10^5^	6.51 × 10^3^	3.51 × 10^5^	1.34 × 10^2^
F1	std	4.69 × 10^5^	6.01 × 10^4^	5.87 × 10^4^	4.34 × 10^6^	2.33 × 10^6^	8.53 × 10^4^	1.60 × 10^6^	1.07 × 10^3^
F1	avg	1.94 × 10^5^	1.62 × 10^5^	1.19 × 10^5^	1.02 × 10^6^	3.73 × 10^6^	7.95 × 10^4^	2.60 × 10^6^	8.23 × 10^2^
F1	median	5.92 × 10^4^	1.60 × 10^5^	1.11 × 10^5^	2.82 × 10^4^	3.28 × 10^6^	4.10 × 10^4^	2.40 × 10^6^	4.37 × 10^2^
F2	min	2.05 × 10^2^	2.30 × 10^2^	2.06 × 10^2^	1.38 × 10^4^	1.13 × 10^5^	2.06 × 10^2^	1.43 × 10^7^	2.00 × 10^2^
F2	std	2.09 × 10^3^	4.49 × 10^2^	2.69 × 10^3^	2.11 × 10^8^	1.41 × 10^5^	1.01 × 10^3^	2.26 × 10^7^	0
F2	avg	2.07 × 10^3^	7.07 × 10^2^	3.38 × 10^3^	6.77 × 10^7^	3.34 × 10^5^	9.86 × 10^2^	4.59 × 10^7^	2.00 × 10^2^
F2	median	1.37 × 10^3^	5.92 × 10^2^	2.85 × 10^3^	3.74 × 10^4^	3.07 × 10^5^	7.00 × 10^2^	4.14 × 10^7^	2.00 × 10^2^
F3	min	3.78 × 10^2^	3.25 × 10^2^	3.04 × 10^2^	3.02 × 10^2^	1.80 × 10^3^	3.99 × 10^2^	2.59 × 10^3^	3.00 × 10^2^
F3	std	3.99 × 10^3^	5.79 × 10^2^	6.02 × 10^1^	2.86 × 10^3^	1.92 × 10^3^	1.37 × 10^3^	7.32 × 10^2^	0
F3	avg	3.58 × 10^3^	9.63 × 10^2^	3.51 × 10^2^	1.27 × 10^3^	4.23 × 10^3^	2.34 × 10^3^	3.69 × 10^3^	3.00 × 10^2^
F3	median	1.76 × 10^3^	7.17 × 10^2^	3.27 × 10^2^	4.93 × 10^2^	3.73 × 10^3^	2.04 × 10^3^	3.63 × 10^3^	3.00 × 10^2^
F4	min	4.00 × 10^2^	4.02 × 10^2^	4.00 × 10^2^	4.01 × 10^2^	4.00 × 10^2^	4.00 × 10^2^	4.21 × 10^2^	4.00 × 10^2^
F4	std	1.48 × 10^1^	1.14 × 10^1^	1.62 × 10^1^	2.92 × 10^1^	3.27 × 10^1^	1.04	1.38 × 10^1^	1.64 × 10^1^
F4	avg	4.23 × 10^2^	4.20 × 10^2^	4.13 × 10^2^	4.31 × 10^2^	4.30 × 10^2^	4.00 × 10^2^	4.44 × 10^2^	4.17 × 10^2^
F4	median	4.35 × 10^2^	4.19 × 10^2^	4.05 × 10^2^	4.35 × 10^2^	4.35 × 10^2^	4.00 × 10^2^	4.43 × 10^2^	4.04 × 10^2^
F5	min	5.20 × 10^2^	5.19 × 10^2^	5.00 × 10^2^	5.20 × 10^2^	5.20 × 10^2^	5.00 × 10^2^	5.20 × 10^2^	5.00 × 10^2^
F5	std	0.06	0.21	6.16	0.07	0.09	4.47	0.07	7.4
F5	avg	5.20 × 10^2^	5.20 × 10^2^	5.18 × 10^2^	5.20 × 10^2^	5.20 × 10^2^	5.19 × 10^2^	5.20 × 10^2^	5.17 × 10^2^
F5	median	5.20 × 10^2^	5.20 × 10^2^	5.20 × 10^2^	5.20 × 10^2^	5.20 × 10^2^	5.20 × 10^2^	5.20 × 10^2^	5.20 × 10^2^
F6	min	6.01 × 10^2^	6.00 × 10^2^	6.00 × 10^2^	6.04 × 10^2^	6.04 × 10^2^	6.00 × 10^2^	6.04 × 10^2^	6.00 × 10^2^
F6	std	1.47	1.3	0.76	1.75	1.66	0.81	0.52	0.21
F6	avg	6.04 × 10^2^	6.02 × 10^2^	6.01 × 10^2^	6.06 × 10^2^	6.08 × 10^2^	6.01 × 10^2^	6.05 × 10^2^	6.00 × 10^2^
F6	median	6.04 × 10^2^	6.02 × 10^2^	6.00 × 10^2^	6.06 × 10^2^	6.07 × 10^2^	6.02 × 10^2^	6.05 × 10^2^	6.00 × 10^2^
F7	min	7.00 × 10^2^	7.00 × 10^2^	7.00 × 10^2^	7.00 × 10^2^	7.01 × 10^2^	7.00 × 10^2^	7.01 × 10^2^	7.00 × 10^2^
F7	std	1.46	0.04	0.02	5.62	0.17	0.05	0.98	0.05
F7	avg	7.01 × 10^2^	7.00 × 10^2^	7.00 × 10^2^	7.02 × 10^2^	7.01 × 10^2^	7.00 × 10^2^	7.02 × 10^2^	7.00 × 10^2^
F7	median	7.00 × 10^2^	7.00 × 10^2^	7.00 × 10^2^	7.01 × 10^2^	7.01 × 10^2^	7.00 × 10^2^	7.02 × 10^2^	7.00 × 10^2^
F8	min	8.06 × 10^2^	8.00 × 10^2^	8.01 × 10^2^	8.06 × 10^2^	8.12 × 10^2^	8.00 × 10^2^	8.13 × 10^2^	8.00 × 10^2^
F8	std	7.22	0	3.21	11.7	7.63	0	4.79	0
F8	avg	8.16 × 10^2^	8.00 × 10^2^	8.05 × 10^2^	8.29 × 10^2^	8.26 × 10^2^	8.00 × 10^2^	8.21 × 10^2^	8.00 × 10^2^
F8	median	8.16 × 10^2^	8.00 × 10^2^	8.04 × 10^2^	8.31 × 10^2^	8.25 × 10^2^	8.00 × 10^2^	8.21 × 10^2^	8.00 × 10^2^
F9	min	9.09 × 10^2^	9.08 × 10^2^	9.03 × 10^2^	9.16 × 10^2^	9.16 × 10^2^	9.03 × 10^2^	9.17 × 10^2^	9.03 × 10^2^
F9	std	9.67	3.26	3.23	11.4	9.84	1.98	4.4	3.26
F9	avg	9.27 × 10^2^	9.13 × 10^2^	9.09 × 10^2^	9.32 × 10^2^	9.39 × 10^2^	9.08 × 10^2^	9.27 × 10^2^	9.08 × 10^2^
F9	median	9.26 × 10^2^	9.13 × 10^2^	9.09 × 10^2^	9.30 × 10^2^	9.40 × 10^2^	9.08 × 10^2^	9.28 × 10^2^	9.07 × 10^2^
F10	min	1.22 × 10^3^	1.00 × 10^3^	1.02 × 10^3^	1.17 × 10^3^	1.07 × 10^3^	1.00 × 10^3^	1.28 × 10^3^	1.00 × 10^3^
F10	std	24.7	6.82	9.56	23.8	21.1	0.08	11.8	1.75
F10	avg	1.57 × 10^3^	1.02 × 10^3^	1.16 × 10^3^	1.63 × 10^3^	1.34 × 10^3^	1.00 × 10^3^	1.50 × 10^3^	1.00 × 10^3^
F10	median	1.58 × 10^3^	1.02 × 10^3^	1.16 × 10^3^	1.63 × 10^3^	1.33 × 10^3^	1.00 × 10^3^	1.49 × 10^3^	1.00 × 10^3^
F11	min	1.53 × 10^3^	1.57 × 10^3^	1.13 × 10^3^	1.38 × 10^3^	1.69 × 10^3^	1.11 × 10^3^	1.52 × 10^3^	1.25 × 10^3^
F11	std	27.2	12.1	18.5	23.7	21.1	11.1	12.7	12.6
F11	avg	1.96 × 10^3^	1.74 × 10^3^	1.41 × 10^3^	1.79 × 10^3^	1.99 × 10^3^	1.34 × 10^3^	1.81 × 10^3^	1.52 × 10^3^
F11	median	1.90 × 10^3^	1.70 × 10^3^	1.36 × 10^3^	1.86 × 10^3^	1.97 × 10^3^	1.32 × 10^3^	1.81 × 10^3^	1.52 × 10^3^
F12	min	1.20 × 10^3^	1.20 × 10^3^	1.20 × 10^3^	1.20 × 10^3^	1.20 × 10^3^	1.20 × 10^3^	1.20 × 10^3^	1.20 × 10^3^
F12	std	0.09	0.09	0.09	0.21	0.26	0.02	0.11	0.2
F12	avg	1.20 × 10^3^	1.20 × 10^3^	1.20 × 10^3^	1.20 × 10^3^	1.20 × 10^3^	1.20 × 10^3^	1.20 × 10^3^	1.20 × 10^3^
F12	median	1.20 × 10^3^	1.20 × 10^3^	1.20 × 10^3^	1.20 × 10^3^	1.20 × 10^3^	1.20 × 10^3^	1.20 × 10^3^	1.20 × 10^3^
F13	min	1.30 × 10^3^	1.30 × 10^3^	1.30 × 10^3^	1.30 × 10^3^	1.30 × 10^3^	1.30 × 10^3^	1.30 × 10^3^	1.30 × 10^3^
F13	std	0.17	0.05	0.03	0.5	0.24	0.06	0.07	0.05
F13	avg	1.30 × 10^3^	1.30 × 10^3^	1.30 × 10^3^	1.30 × 10^3^	1.30 × 10^3^	1.30 × 10^3^	1.30 × 10^3^	1.30 × 10^3^
F13	median	1.30 × 10^3^	1.30 × 10^3^	1.30 × 10^3^	1.30 × 10^3^	1.30 × 10^3^	1.30 × 10^3^	1.30 × 10^3^	1.30 × 10^3^
F14	min	1.40 × 10^3^	1.40 × 10^3^	1.40 × 10^3^	1.40 × 10^3^	1.40 × 10^3^	1.40 × 10^3^	1.40 × 10^3^	1.40 × 10^3^
F14	std	0.28	0.07	0.06	4.38	0.28	0.08	0.06	0.07
F14	avg	1.40 × 10^3^	1.40 × 10^3^	1.40 × 10^3^	1.40 × 10^3^	1.40 × 10^3^	1.40 × 10^3^	1.40 × 10^3^	1.40 × 10^3^
F14	median	1.40 × 10^3^	1.40 × 10^3^	1.40 × 10^3^	1.40 × 10^3^	1.40 × 10^3^	1.40 × 10^3^	1.40 × 10^3^	1.40 × 10^3^
F15	min	1.50 × 10^3^	1.50 × 10^3^	1.50 × 10^3^	1.50 × 10^3^	1.50 × 10^3^	1.50 × 10^3^	1.50 × 10^3^	1.50 × 10^3^
F15	std	0.49	0.29	0.3	10.6	2.8	0.3	0.92	0.31
F15	avg	1.50 × 10^3^	1.50 × 10^3^	1.50 × 10^3^	1.51 × 10^3^	1.51 × 10^3^	1.50 × 10^3^	1.51 × 10^3^	1.50 × 10^3^
F15	median	1.50 × 10^3^	1.50 × 10^3^	1.50 × 10^3^	1.50 × 10^3^	1.51 × 10^3^	1.50 × 10^3^	1.51 × 10^3^	1.50 × 10^3^
F16	min	1.60 × 10^3^	1.60 × 10^3^	1.60 × 10^3^	1.60 × 10^3^	1.60 × 10^3^	1.60 × 10^3^	1.60 × 10^3^	1.60 × 10^3^
F16	std	0.5	0.16	0.64	0.42	0.17	0.33	0.13	0.38
F16	avg	1.60 × 10^3^	1.60 × 10^3^	1.60 × 10^3^	1.60 × 10^3^	1.60 × 10^3^	1.60 × 10^3^	1.60 × 10^3^	1.60 × 10^3^
F16	median	1.60 × 10^3^	1.60 × 10^3^	1.60 × 10^3^	1.60 × 10^3^	1.60 × 10^3^	1.60 × 10^3^	1.60 × 10^3^	1.60 × 10^3^
F17	min	1.78 × 10^3^	8.32 × 10^3^	1.75 × 10^3^	1.78 × 10^3^	4.59 × 10^3^	2.26 × 10^3^	3.50 × 10^3^	1.71 × 10^3^
F17	std	3.09 × 10^3^	3.74 × 10^4^	1.55 × 10^3^	2.86 × 10^3^	2.41 × 10^4^	8.86 × 10^4^	9.47 × 10^3^	3.16 × 10^1^
F17	avg	4.58 × 10^3^	4.03 × 10^4^	3.19 × 10^3^	3.69 × 10^3^	2.55 × 10^4^	4.72 × 10^4^	1.60 × 10^4^	1.75 × 10^3^
F17	median	3.28 × 10^3^	3.18 × 10^4^	2.54 × 10^3^	2.42 × 10^3^	1.43 × 10^4^	1.60 × 10^4^	1.50 × 10^4^	1.74 × 10^3^
F18	min	1.93 × 10^3^	2.00 × 10^3^	1.92 × 10^3^	1.85 × 10^3^	2.76 × 10^3^	1.80 × 10^3^	2.65 × 10^3^	1.80 × 10^3^
F18	std	7.43 × 10^3^	2.41 × 10^3^	3.22 × 10^3^	4.79 × 10^1^	8.54 × 10^3^	2.45 × 10^3^	2.36 × 10^3^	1.06
F18	avg	1.09 × 10^4^	4.66 × 10^3^	4.30 × 10^3^	1.92 × 10^3^	1.29 × 10^4^	3.04 × 10^3^	6.63 × 10^3^	1.80 × 10^3^
F18	median	1.13 × 10^4^	4.70 × 10^3^	2.78 × 10^3^	1.91 × 10^3^	1.25 × 10^4^	1.97 × 10^3^	7.57 × 10^3^	1.80 × 10^3^
F19	min	1.90 × 10^3^	1.90 × 10^3^	1.90 × 10^3^	1.90 × 10^3^	1.90 × 10^3^	1.90 × 10^3^	1.90 × 10^3^	1.90 × 10^3^
F19	std	1.04	0.25	0.33	1.7	1.11	0.32	0.43	0.2
F19	avg	1.90 × 10^3^	1.90 × 10^3^	1.90 × 10^3^	1.90 × 10^3^	1.90 × 10^3^	1.90 × 10^3^	1.90 × 10^3^	1.90 × 10^3^
F19	median	1.90 × 10^3^	1.90 × 10^3^	1.90 × 10^3^	1.90 × 10^3^	1.90 × 10^3^	1.90 × 10^3^	1.90 × 10^3^	1.90 × 10^3^
F20	min	2.04 × 10^3^	2.01 × 10^3^	2.00 × 10^3^	2.02 × 10^3^	2.24 × 10^3^	2.00 × 10^3^	2.21 × 10^3^	2.00 × 10^3^
F20	std	3.59 × 10^3^	7.93 × 10^2^	1.54 × 10^2^	5.27 × 10^1^	4.01 × 10^3^	2.11 × 10^3^	1.07 × 10^3^	0.39
F20	avg	4.91 × 10^3^	2.69 × 10^3^	2.09 × 10^3^	2.10 × 10^3^	7.94 × 10^3^	3.03 × 10^3^	3.60 × 10^3^	2.00 × 10^3^
F20	median	3.40 × 10^3^	2.37 × 10^3^	2.05 × 10^3^	2.07 × 10^3^	7.26 × 10^3^	2.42 × 10^3^	3.28 × 10^3^	2.00 × 10^3^
F21	min	2.35 × 10^3^	2.20 × 10^3^	2.13 × 10^3^	2.20 × 10^3^	3.23 × 10^3^	2.29 × 10^3^	3.88 × 10^3^	2.10 × 10^3^
F21	std	1.09 × 10^3^	1.24 × 10^3^	1.13 × 10^2^	1.97 × 10^2^	8.14 × 10^3^	1.19 × 10^4^	2.21 × 10^3^	4.9
F21	avg	3.63 × 10^3^	3.66 × 10^3^	2.28 × 10^3^	2.48 × 10^3^	9.78 × 10^3^	1.06 × 10^4^	6.33 × 10^3^	2.10 × 10^3^
F21	median	3.53 × 10^3^	3.45 × 10^3^	2.26 × 10^3^	2.43 × 10^3^	7.92 × 10^3^	5.16 × 10^3^	5.59 × 10^3^	2.10 × 10^3^
F22	min	2.23 × 10^3^	2.20 × 10^3^	2.20 × 10^3^	2.22 × 10^3^	2.23 × 10^3^	2.20 × 10^3^	2.23 × 10^3^	2.20 × 10^3^
F22	std	7.32 × 10^1^	2.3	9.45	4.87 × 10^1^	9.80 × 10^1^	3.64 × 10^1^	9.25	3.72
F22	avg	2.35 × 10^3^	2.20 × 10^3^	2.22 × 10^3^	2.26 × 10^3^	2.34 × 10^3^	2.21 × 10^3^	2.24 × 10^3^	2.20 × 10^3^
F22	median	2.36 × 10^3^	2.20 × 10^3^	2.22 × 10^3^	2.24 × 10^3^	2.35 × 10^3^	2.20 × 10^3^	2.24 × 10^3^	2.20 × 10^3^
F23	min	2.63 × 10^3^	2.63 × 10^3^	2.63 × 10^3^	2.50 × 10^3^	2.50 × 10^3^	2.63 × 10^3^	2.50 × 10^3^	2.50 × 10^3^
F23	std	2.9	0	0	0	0	0	6.00 × 10^1^	0
F23	avg	2.63 × 10^3^	2.63 × 10^3^	2.63 × 10^3^	2.50 × 10^3^	2.50 × 10^3^	2.63 × 10^3^	2.54 × 10^3^	2.50 × 10^3^
F23	median	2.63 × 10^3^	2.63 × 10^3^	2.63 × 10^3^	2.50 × 10^3^	2.50 × 10^3^	2.63 × 10^3^	2.50 × 10^3^	2.50 × 10^3^
F24	min	2.52 × 10^3^	2.51 × 10^3^	2.51 × 10^3^	2.52 × 10^3^	2.54 × 10^3^	2.51 × 10^3^	2.52 × 10^3^	2.51 × 10^3^
F24	std	2.53 × 10^1^	3.38	1.92 × 10^1^	2.87 × 10^1^	2.18 × 10^1^	4.75	6.43	3.17
F24	avg	2.55 × 10^3^	2.52 × 10^3^	2.52 × 10^3^	2.56 × 10^3^	2.59 × 10^3^	2.52 × 10^3^	2.54 × 10^3^	2.51 × 10^3^
F24	median	2.55 × 10^3^	2.52 × 10^3^	2.52 × 10^3^	2.56 × 10^3^	2.60 × 10^3^	2.52 × 10^3^	2.54 × 10^3^	2.51 × 10^3^
F25	min	2.64 × 10^3^	2.64 × 10^3^	2.62 × 10^3^	2.64 × 10^3^	2.66 × 10^3^	2.62 × 10^3^	2.65 × 10^3^	2.62 × 10^3^
F25	std	1.82 × 10^1^	1.85 × 10^1^	3.04 × 10^1^	1.44 × 10^1^	9.06	3.41 × 10^1^	2.23 × 10^1^	3.18 × 10^1^
F25	avg	2.69 × 10^3^	2.67 × 10^3^	2.68 × 10^3^	2.70 × 10^3^	2.70 × 10^3^	2.66 × 10^3^	2.68 × 10^3^	2.66 × 10^3^
F25	median	2.70 × 10^3^	2.67 × 10^3^	2.70 × 10^3^	2.70 × 10^3^	2.70 × 10^3^	2.65 × 10^3^	2.67 × 10^3^	2.64 × 10^3^
F26	min	2.70 × 10^3^	2.70 × 10^3^	2.70 × 10^3^	2.70 × 10^3^	2.70 × 10^3^	2.70 × 10^3^	2.70 × 10^3^	2.70 × 10^3^
F26	std	2.23 × 10^1^	0.04	0.04	0.08	0.16	0.06	0.06	0.04
F26	avg	2.71 × 10^3^	2.70 × 10^3^	2.70 × 10^3^	2.70 × 10^3^	2.70 × 10^3^	2.70 × 10^3^	2.70 × 10^3^	2.70 × 10^3^
F26	median	2.70 × 10^3^	2.70 × 10^3^	2.70 × 10^3^	2.70 × 10^3^	2.70 × 10^3^	2.70 × 10^3^	2.70 × 10^3^	2.70 × 10^3^
F27	min	2.75 × 10^3^	2.71 × 10^3^	2.70 × 10^3^	2.70 × 10^3^	2.90 × 10^3^	2.70 × 10^3^	2.70 × 10^3^	2.70 × 10^3^
F27	std	8.78 × 10^1^	1.57 × 10^2^	1.90 × 10^2^	7.13 × 10^1^	0	1.28 × 10^2^	1.61 × 10^2^	1.36 × 10^2^
F27	avg	3.08 × 10^3^	2.93 × 10^3^	2.95 × 10^3^	2.87 × 10^3^	2.90 × 10^3^	3.00 × 10^3^	2.79 × 10^3^	2.79 × 10^3^
F27	median	3.10 × 10^3^	3.02 × 10^3^	3.07 × 10^3^	2.90 × 10^3^	2.90 × 10^3^	3.04 × 10^3^	2.71 × 10^3^	2.70 × 10^3^
F28	min	3.19 × 10^3^	3.17 × 10^3^	3.16 × 10^3^	3.00 × 10^3^	3.00 × 10^3^	3.16 × 10^3^	3.18 × 10^3^	3.00 × 10^3^
F28	std	2.30 × 10^2^	4.9	6.42 × 10^1^	0	0	4.08 × 10^1^	4.56 × 10^1^	4.86 × 10^1^
F28	avg	3.43 × 10^3^	3.18 × 10^3^	3.22 × 10^3^	3.00 × 10^3^	3.00 × 10^3^	3.22 × 10^3^	3.24 × 10^3^	3.02 × 10^3^
F28	median	3.35 × 10^3^	3.18 × 10^3^	3.17 × 10^3^	3.00 × 10^3^	3.00 × 10^3^	3.21 × 10^3^	3.23 × 10^3^	3.00 × 10^3^
F29	min	3.18 × 10^3^	3.41 × 10^3^	3.21 × 10^3^	3.10 × 10^3^	3.10 × 10^3^	3.13 × 10^3^	3.27 × 10^3^	3.09 × 10^3^
F29	std	1.39 × 10^6^	2.20 × 10^2^	5.82 × 10^5^	5.48 × 10^2^	8.19 × 10^5^	6.91 × 10^1^	7.42 × 10^1^	8.92
F29	avg	7.23 × 10^5^	3.60 × 10^3^	1.92 × 10^5^	3.56 × 10^3^	1.88 × 10^5^	3.23 × 10^3^	3.38 × 10^3^	3.12 × 10^3^
F29	median	3.37 × 10^3^	3.54 × 10^3^	3.40 × 10^3^	3.35 × 10^3^	3.69 × 10^3^	3.22 × 10^3^	3.37 × 10^3^	3.13 × 10^3^
F30	min	3.71 × 10^3^	3.47 × 10^3^	3.49 × 10^3^	3.54 × 10^3^	4.06 × 10^3^	3.52 × 10^3^	3.67 × 10^3^	3.55 × 10^3^
F30	std	5.31 × 10^2^	4.81 × 10^1^	1.63 × 10^2^	6.95 × 10^2^	8.70 × 10^2^	1.01 × 10^2^	3.58 × 10^2^	5.66 × 10^1^
F30	avg	4.62 × 10^3^	3.54 × 10^3^	3.61 × 10^3^	4.39 × 10^3^	5.51 × 10^3^	3.68 × 10^3^	4.40 × 10^3^	3.62 × 10^3^
F30	median	4.61 × 10^3^	3.52 × 10^3^	3.55 × 10^3^	4.25 × 10^3^	5.42 × 10^3^	3.65 × 10^3^	4.35 × 10^3^	3.62 × 10^3^

**Table 2:** Differential performance and average rank of CEC2014.

Algorithm	IESOA	ESOA	DOA	HHO	BKA	SBOA	DE	PSO
Differential expression (Y/N)	0/0	10/3	11/1	12/0	12/0	12/0	12/0	12/0
Average rank	1.83	5.87	3.23	6.6	5.4	3.03	3.97	5.93

**Table 3:** The result of standard functions of CEC2019 for different algorithms.

		PSO	DE	SBOA	BKA	HHO	DOA	ESOA	IESOA
F1	min	1.88 × 10^3^	1.13 × 10^6^	1	1	1	6.46 × 10^4^	1	1
F1	std	1.83 × 10^5^	1.57 × 10^6^	1.56 × 10^4^	0	0	3.28 × 10^5^	0	0
F1	avg	1.06 × 10^5^	3.32 × 10^6^	5.81 × 10^3^	1	1	4.34 × 10^5^	1	1
F1	median	3.88 × 10^4^	3.15 × 10^6^	1.65 × 10^1^	1	1	3.43 × 10^5^	1	1
F2	min	5.70 × 10^1^	2.27 × 10^3^	4.14	4.25	4.65	8.30 × 10^1^	4.27	4.2
F2	std	1.50 × 10^2^	4.64 × 10^2^	1.11 × 10^2^	0.27	0.13	1.32 × 10^2^	0.21	0.04
F2	avg	3.26 × 10^2^	3.17 × 10^3^	1.13 × 10^2^	4.47	4.93	3.32 × 10^2^	4.4	4.27
F2	median	3.34 × 10^2^	3.12 × 10^3^	7.08 × 10^1^	4.33	5	3.40 × 10^2^	4.32	4.26
F3	min	1.41	5.13	1	1.41	2.69	1	4.34	1.49
F3	std	1.66	0.79	0.09	1.41	1.64	0.14	0.64	0.68
F3	avg	3.29	6.9	1.39	2.09	4.55	1.38	6.13	3.03
F3	median	3.39	6.97	1.41	1.41	4.16	1.41	6.16	3.24
F4	min	7.96	7.49	2.99	1.20 × 10^1^	1.55 × 10^1^	1	1.45 × 10^1^	4.02
F4	std	1.24 × 10^1^	2.34	2.87	1.76 × 10^1^	1.43 × 10^1^	3.8	8.41	1.99
F4	avg	2.99 × 10^1^	1.28 × 10^1^	8.96	4.11 × 10^1^	4.82 × 10^1^	7.77	3.09 × 10^1^	7.01
F4	median	2.99 × 10^1^	1.30 × 10^1^	9.49	4.30 × 10^1^	4.91 × 10^1^	6.47	3.19 × 10^1^	7.08
F5	min	1.01	1.04	1.01	1.19	1.52	1.01	2.34	1
F5	std	0.07	0.04	0.03	1.15 × 10^1^	0.23	0.06	0.61	0.04
F5	avg	1.12	1.11	1.04	5.92	1.92	1.08	3.15	1.04
F5	median	1.1	1.11	1.03	1.64	1.9	1.07	3.17	1.03
F6	min	1.04	1	1	3.94	3.69	1.03	4.06	1
F6	std	1.2	0.45	0.73	1.77	2.38	0.6	0.73	0
F6	avg	3.36	1.57	1.33	6.44	7.83	1.62	5.65	1
F6	median	3.43	1.48	1	6.5	8.08	1.48	5.62	1
F7	min	4.75 × 10^2^	4.32 × 10^2^	1.91 × 10^1^	3.70 × 10^2^	3.70 × 10^2^	2.18 × 10^2^	7.85 × 10^2^	1.22 × 10^1^
F7	std	2.91 × 10^2^	9.69 × 10^1^	2.77 × 10^2^	2.44 × 10^2^	3.99 × 10^2^	1.27 × 10^2^	1.07 × 10^2^	2.13 × 10^2^
F7	avg	1.00 × 10^3^	5.87 × 10^2^	4.47 × 10^2^	9.34 × 10^2^	1.13 × 10^3^	3.52 × 10^2^	9.47 × 10^2^	4.52 × 10^2^
F7	median	9.61 × 10^2^	5.84 × 10^2^	3.70 × 10^2^	9.36 × 10^2^	1.15 × 10^3^	3.53 × 10^2^	9.11 × 10^2^	4.94 × 10^2^
F8	min	3.33	3.43	2.21	3.55	3.98	2.35	4.03	2.91
F8	std	0.49	0.19	0.46	0.34	0.3	0.36	0.17	0.26
F8	avg	4.13	3.79	3.02	4.2	4.65	3.14	4.31	3.61
F8	median	4.21	3.78	3.04	4.25	4.76	3.16	4.33	3.68
F9	min	1.06	1.13	1.03	1.06	1.2	1.08	1.16	1.11
F9	std	0.13	0.04	0.03	0.1	0.21	0.05	0.08	0.04
F9	avg	1.24	1.24	1.08	1.27	1.45	1.18	1.32	1.21
F9	median	1.23	1.24	1.08	1.26	1.39	1.17	1.35	1.21
F10	min	2.10 × 10^1^	2.11 × 10^1^	1	6.1	2.10 × 10^1^	1.01	1.49 × 10^1^	1
F10	std	0.04	0.03	4.49	3.37	0.07	6.15	1.94	7.38
F10	avg	2.10 × 10^1^	2.11 × 10^1^	1.99 × 10^1^	2.04 × 10^1^	2.11 × 10^1^	1.90 × 10^1^	2.01 × 10^1^	1.82 × 10^1^
F10	median	2.10 × 10^1^	2.11 × 10^1^	2.10 × 10^1^	2.12 × 10^1^	2.10 × 10^1^	2.10 × 10^1^	2.13 × 10^1^	2.12 × 10^1^

**Table 4:** Differential performance and average rank of CEC2019.

Algorithm	IESOA	ESOA	DOA	HHO	BKA	SBOA	DE	PSO
Differential expression (Y/N)	0/0	11/1	12/0	12/1	12/0	12/0	12/1	12/0
Average rank	1.9	5.3	3.1	6.4	5.1	2.6	5.5	5.5

**Table 5:** Comparison table of optimization results for reinforced concrete beam design.

Reinforced Concrete Beam	PSO	DE	SBOA	BKA	HHO	DOA	ESOA	IESOA
worst	158.805	158.805	158.805	158.805	158.816	160.163	158.818	158.805
best	166.079	158.805	158.805	158.817	159.032	174.691	159.372	158.805
std	3.756	0.000	0.000	0.005	0.083	4.848	0.167	0.000
mean	161.715	158.805	158.805	158.809	158.931	168.228	158.941	158.805
median	158.805	158.805	158.805	158.807	158.945	169.004	158.884	158.805

**Table 6:** Comparison table of optimization results for welded beam design.

Welded Beam	PSO	DE	SBOA	BKA	HHO	DOA	ESOA	IESOA
worst	1.6702	1.6985	1.6702	1.6712	1.7618	2.3052	1.7337	1.6702
best	2.5320	2.0256	1.6702	2.3589	2.1838	3.4175	1.9298	1.6702
std	0.2766	0.1328	0.0000	0.2854	0.1261	0.3091	0.0541	0.0000
mean	1.8374	1.8358	1.6702	1.8241	1.8583	2.7175	1.8150	1.6702
median	1.7068	1.7890	1.6702	1.6728	1.8243	2.7223	1.8176	1.6702

**Table 7:** Comparison table of optimization results for pressure vessel design.

Pressure Vessel Design	PSO	DE	SBOA	BKA	HHO	DOA	ESOA	IESOA
worst	6059.7143	6090.6910	6059.7144	6059.7254	6100.7101	6958.6173	6208.2013	6059.7143
best	7332.8415	7332.8415	6059.7259	7944.6878	6979.1412	8874.2681	6622.2110	6090.5262
std	443.5429	385.6625	0.0042	515.8229	266.6043	654.3581	143.7674	14.8835
mean	6558.8130	6318.0520	6059.7178	6896.1340	6566.6105	7901.9759	6412.9297	6068.9579
median	6410.0868	6116.0920	6059.7158	6820.7599	6481.8625	7742.0662	6374.4784	6059.7143

## Data Availability

The data that support the findings of this study are available from the corresponding author upon request. There are no restrictions on data availability.
